# Simulation of the In Vivo Fate of Polymeric Nanoparticles Traced by Environment-Responsive Near-Infrared Dye: A Physiologically Based Pharmacokinetic Modelling Approach

**DOI:** 10.3390/molecules26051271

**Published:** 2021-02-26

**Authors:** Lei Li, Haisheng He, Sifang Jiang, Jianping Qi, Yi Lu, Ning Ding, Hai-Shu Lin, Wei Wu, Xiaoqiang Xiang

**Affiliations:** 1 Department of Clinical Pharmacy and Pharmacy Administration, School of Pharmacy, Fudan University, Shanghai 201203, China; 15211030046@fudan.edu.cn; 2 Key Laboratory of Smart Drug Delivery of MOE and PLA, School of Pharmacy, Fudan University, Shanghai 201203, China; he_haisheng@fudan.edu.cn (H.H.); qijianping@fudan.edu.cn (J.Q.); fd_luyi@fudan.edu.cn (Y.L.); 3 West China School of Pharmacy, Sichuan University, Chengdu 610041, China; sifangjiang@scu.edu.cn; 4 Department of Medicinal Chemistry, School of Pharmacy, Fudan University, Shanghai 201203, China; dingning@fudan.edu.cn; 5 College of Pharmacy, Shenzhen Technology University, Shenzhen 518118, China; linhaishu@sztu.edu.cn

**Keywords:** physiologically based pharmacokinetic model, methoxy poly (ethylene glycol)-poly (ε-caprolactone), nanoparticles, phagocytosis, biodistribution

## Abstract

The application of physiologically based pharmacokinetic models to nanoparticles is still very restricted and challenging, owing to the complicated in vivo transport mechanisms involving nanoparticles, including phagocytosis, enhanced permeability and retention effects, cellular recognition, and internalisation, enzymatic degradation, lymphatic transport, and changes in physical properties. In our study, five nanoparticle formulations were synthesised using polycaprolactone as a framework material and methoxy poly (ethylene glycol)-poly(ε-caprolactone) as a long-circulating decorating material, as well as types of environmentally responsive near-infrared aza-boron-dipyrromethene dyes. According to quantification data and direct visualisation involving specific organs, a phagocytosis physiologically based pharmacokinetic model was developed to describe the dynamics of nanoparticles within and between organs in mice, considering cellular mechanisms involving phagocytosis and enhanced permeability and retention effects. Our results offer a better understanding of the in vivo fate of polymeric nanoparticles.

## 1. Introduction

Nanoparticles (NPs) have been widely employed in drug delivery systems (DDS) because they can increase drug absorption and bioavailability by enhancing drug dissolution rates and improving selective uptake in certain tissues. As important as DDS, organic polymeric methoxy poly (ethylene glycol)-poly (ε-caprolactone) (mPEG-PCL) NPs have attracted considerable attention due to their characteristics involving amphiphilicity, biodegradability, and excellent biocompatibility [1]. Taking advantage of enhanced permeability and retention (EPR) effects [2], many NPs synthesised by mPEG-PCL copolymers have been designed to deliver antineoplastic drugs to tumours. However, only a very small number of mPEG-PCL NP formulations have entered clinical research phases, mainly because few studies have evaluated the biodistribution of mPEG-PCL NPs in vivo [1]. Furthermore, it has been estimated that, on average, only 0.7% of NP doses can enter tumours [3]. Therefore, it is important to understand the in vivo behaviour of NPs to assess their efficacy and toxicity. Physiologically based pharmacokinetic (PBPK) modelling can be a valuable tool for describing, predicting, and simulating the in vivo absorption, distribution, metabolism, and excretion (ADME) of NPs. Based on the physiological values and anatomical features integrated by a system of mathematical equations, these models can not only predict and quantify drug exposure in blood and each organ, but also compare data from different administration routes and scale-up such data from animals to humans [4].

PBPK modelling is a useful tool for dealing with various types of NPs such as quantum dots (QDs) [5], silver NPs [6], gold/dendrimer composite NPs [7], and poly (lactic-co-glycolic) acid (PLGA) NPs [8]. For example, a model of titanium dioxide (TiO_2_) NPs has been extrapolated from animals to humans and has been demonstrated to be useful in assessing the risk of ingesting nano-TiO_2_ [9]. Early models did not address cellular phagocytosis, which is an important process affecting the behaviour of NPs in the body [10]. However, a number of recent models have begun to consider cellular phagocytosis [5,7]. Liang et al. developed a PBPK model to characterise and predict the in vivo behaviour of long-circulating inorganic QD NPs (QD/NPs), suggesting that the biodistribution of QD/NPs is largely determined by the uptake and release of phagocytic cells (PCs) by targeted organs [5]. Compared with PBPK models of inorganic metallic NPs and QD/NPs, much less research involving organic polymeric NPs and cellular phagocytosis has been reported. To date, there have been only a small number of reports concerning polymeric NPs and mechanisms involved in cellular phagocytosis [7,11,12,13,14,15]. For example, Li et al. evaluated the biodistribution of polyethylene glycol-coated polyacrylamide (PEG/PAA) NPs using a PBPK model based on the mechanism of cellular phagocytosis, which indicated that the phagocytosing cell (PC) system greatly affected the pharmacokinetic behaviour of PEG/PAA NPs [11].

According to previous studies, when developing and validating PBPK models of NPs, one of the main challenges is the lack of rapid and reliable analytical approaches [16,17]. This is because the ADME processes involving NPs are much more complex than those of small molecules. In addition, it is much more difficult to isolate NPs from tissues than from plasma samples. In contrast to radioactivity measurements and traditional isolation methods, in our study, we developed a novel type of NP using environmentally responsive near-infrared (NIR) aza-boron-dipyrromethene (aza-BODIPY) dyes, as shortened to P2 [18]. A distinctive characteristic of P2-labelled mPEG-PCL NPs is their ability to emit fluorescence with an integral state of NPs, and in quenching fluorescence immediately after the P2 dye is released from the NPs and makes contact with water. By tracking the signals of P2, we were able to explore the in vivo fate of integral NPs. Here, we synthesised five mPEG-PCL NP formulations with different sizes, different ratios of mPEG-PCL/PCL, and different weights of mPEG coating. Next, a PBPK model for the five mPEG-PCL NP formulations was successfully generated by directly fitting the observed concentration data at the organ level in mice. This PBPK model of NPs can be used to quantitatively describe and predict concentration–time profiles and exposure of the five NP formulations in blood and individual organs. Our results showed that the PBPK model was accurate, reliable, and highly predictive of the in vivo pharmacokinetics of NPs, and could be potentially used to guide NP design and extrapolate pharmacokinetic data obtained in animals to those in humans. Our findings suggested that PBPK modelling offers a superior understanding of the in vivo fate of polymeric NPs through the comparison of key parameters in such PBPK models.

## 2. Results

### 2.1. Preparation and Characterisation of NPs

We synthesised five different types of mPEG-PCL NPs by varying the mPEG-PCL molar ratio and PEG molecular weight, namely mPEG5k-9.09%-80 nm NPs, mPEG5k-28.57%-80 nm NPs, mPEG5k-9.09%-200 nm NPs, mPEG5k-28.57%-200 nm NPs, and mPEG2k-28.57%-200 nm NPs (Table S1). The size distributions of various mPEG-NPs encapsulating P2 are shown in Figure S1.

### 2.2. Live Imaging of mPEG-PCL NPs

**Figure 1:**
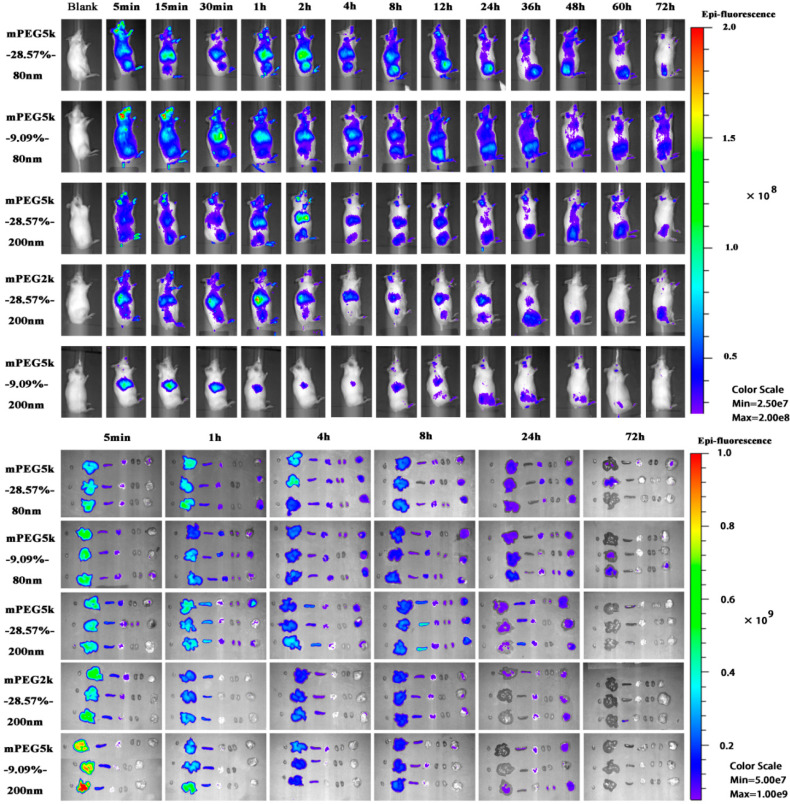
Live imaging of methoxy poly (ethylene glycol)-poly (ε-caprolactone) (mPEG-PCL) nanoparticles (NPs) after intravenous administration and ex vivo live imaging of organs dissected from tumour-bearing mice.

The distribution of five mPEG-PCL NP formulations in tumour-bearing mice in vivo and each organ in vitro were observed after intravenous administration (Figure 1). The fluorescence signals of the five formulations in vivo showed that the liver and spleen were the two organs with the strongest fluorescence signals, and the residence time of the NPs in the tumour tissue was as long as 72 h. In the mPEG5K-9.09%-200 nm group, abundant fluorescence signals were accumulated in the liver by 5 min, indicating that the NPs were rapidly taken up by the liver after entering the bloodstream. The fluorescent signals at tumour sites were always weak, indicating that a small quantity of NPs were delivered to the tumour. The fluorescent signal in the liver of the PEG5K-9.09%-200 nm group mice was stronger than that in the mPEG5K-9.09%-80 nm and mPEG5K-28.57%-200 nm groups, whereas the signal in tumour tissues was weaker than that in the other two groups. In the mPEG5K-28.57%-200 nm cohort, the tumour signal was higher than that in the mPEG2K-28.57%-200 nm group. All of these results indicated that the degree of PEG modification, PEG chain length, and NP size affected the delivery efficiency of NPs to the tumour. However, the results of living body distribution can only provide an approximate understanding of the distribution of particles in an animal body (Figure S2) and cannot accurately reflect the specific distribution of particles in various organs. Thus, we used PBPK modelling to quantitatively describe the distribution of NPs in vivo by calculating and evaluating relevant parameters in the PBPK model.

### 2.3. PBPK Model

Both the PCs-PBPK model and EPR-PBPK model were generated in this study and goodness of fit was evaluated to determine how well the experimental data could be simulated via the R^2^ values. For all five formulations, the PCs-PBPK model performed better than the EPR-PBPK model (Table 1). As a result, the PCs-PBPK model was selected for all other work described in this study, and the simulations involving the EPR-PBPK model are provided in the Supporting information.

**Figure 2:**
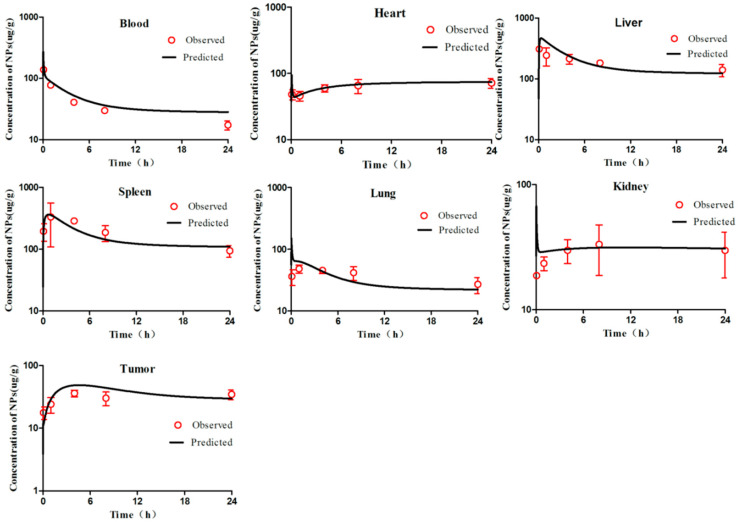
Simulated and experimentally measured concentration–time curves of mPEG5k-9.09%-80 nm NPs in murine blood and tissues after intravenous injection. Simulation results are represented by solid lines in each panel, and the mean values of measured data are represented by red dots. Error bars represent standard deviation of experimentally measured data.

**Figure 3:**
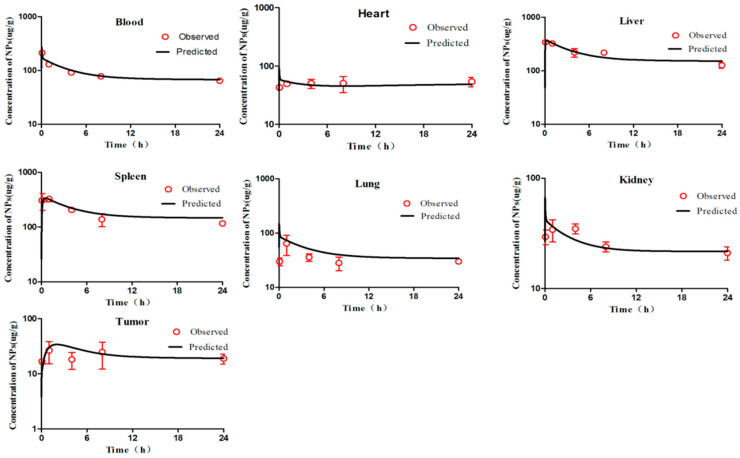
Simulated and experimentally measured concentration–time curves of mPEG5k-28.57%-80 nm NPs in mouse blood and tissues after intravenous injection.

**Figure 4:**
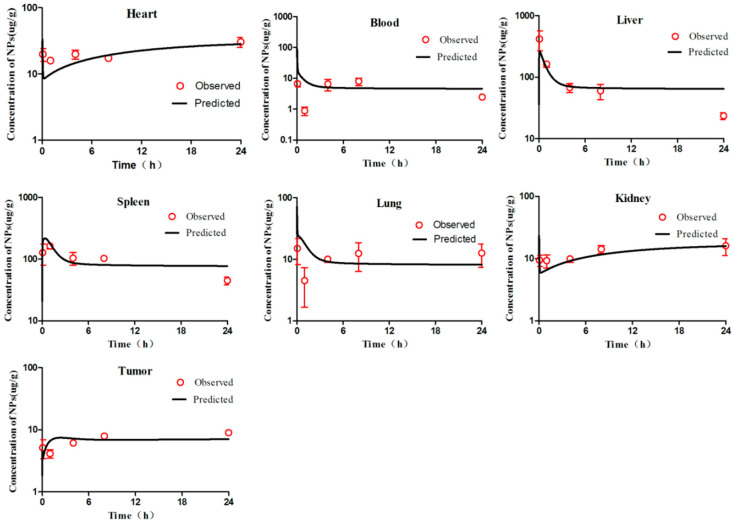
Simulated and experimentally measured concentration–time curves of mPEG5k-9.09%-200 nm NPs in murine blood and tissues after intravenous injection.

**Figure 5:**
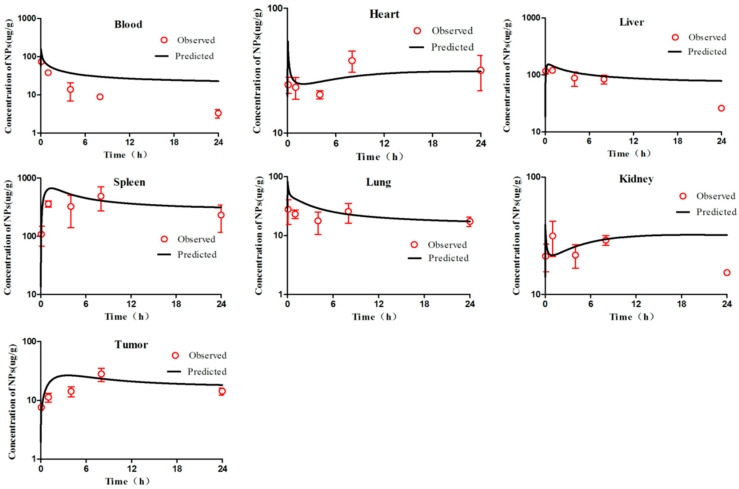
Simulated and experimentally measured concentration–time curves of mPEG5k-28.57%-200 nm NPs in murine blood and tissues after intravenous injection.

**Figure 6:**
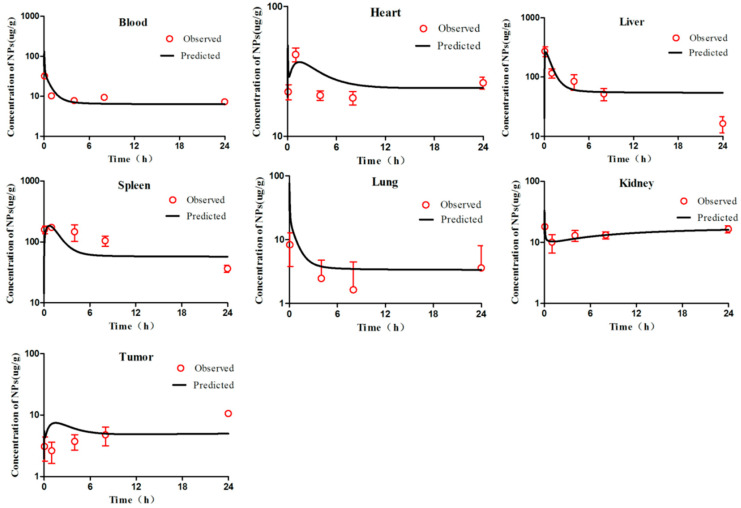
Simulated and experimentally measured concentration–time curves of mPEG2k-28.57%-200 nm NPs in mouse blood and tissues after intravenous injection.

According to the WHO criteria for PBPK model evaluation, it is considered “acceptable” when discrepancies between simulated and observed data are <two-fold. The simulation results (Figure 2, Figure 3, Figure 4, Figure 5 and Figure 6) suggested that the pharmacokinetic profiles of all five mPEG-PCL NP formulations were well simulated in the blood and tissue using the PBPK model. The linear regression coefficients (R^2^) were 0.8810, 0.9068, 0.7755, 0.8091, and 0.8561 for mPEG5k-9.09%-80 nm NPs, mPEG5k-28.57%-80 nm NPs, mPEG5k-9.09%-200 nm NPs, mPEG5k-28.57%-200 nm NPs, and mPEG2k-28.57%-200 nm NPs (Figure S3), respectively, indicating an excellent goodness-of-fit of the PCs-PBPK model.

### 2.4. Simulation Results

With a slight (>two-fold) overestimation of the plasma concentrations of mPEG5k-9.09%-200 nm NPs and mPEG2k-28.57%-200 nm NPs, the general simulation results of the PBPK model fitted well with the experimental concentrations of NPs in blood, heart, liver, spleen, lung, kidney, and tumours of mice after intravenous injection (i.v.) of mPEG5k-9.09%-80 nm NPs (Figure 2), mPEG5k-28.57%-80 nm NPs (Figure 3), mPEG5k-9.09%-200 nm NPs (Figure 4), mPEG5k-28.57%-200 nm NPs (Figure 5), and mPEG2k-28.57%-200 nm NPs (Figure 6), respectively. According to the simulation results presented in Figure 2, after the NPs entered the body, they were distributed more into the bloodstream and certain tissues such as liver and spleen, but less so in heart, kidney, lung, and tumour tissues, which was consistent with the higher K_max_ (h^−1^). For example, in the model for mPEG5k-9.09%-80 nm NPs, the K_max_ (h^−1^) values for liver and spleen were 151 h^−1^ and 83 h^−1^, respectively, while those for heart, kidney, and lung were 0.16 h^−1^ 0.05 h^−1^ 0.05 h^−1^ and 15 h^−1^, respectively. In addition, we found that the 80 nm mPEG-PCL NP concentration in blood was higher than that of 200 nm mPEG-PCL NPs. Furthermore, the concentration of NPs containing more mPEG-PCL, such as mPEG5k-28.57%-80 nm in blood, was higher than that of animals with less PEG, such as mPEG5k-9.09%-80 nm.

The concentration–time profiles of mPEG-PCL NPs in tissues and plasma were fitted simultaneously to obtain mPEG-PCL NP and tissue-specific parameters. All parameters of the PBPK model are listed in Table 2.

### 2.5. Sensitive Analysis

**Figure 7:**
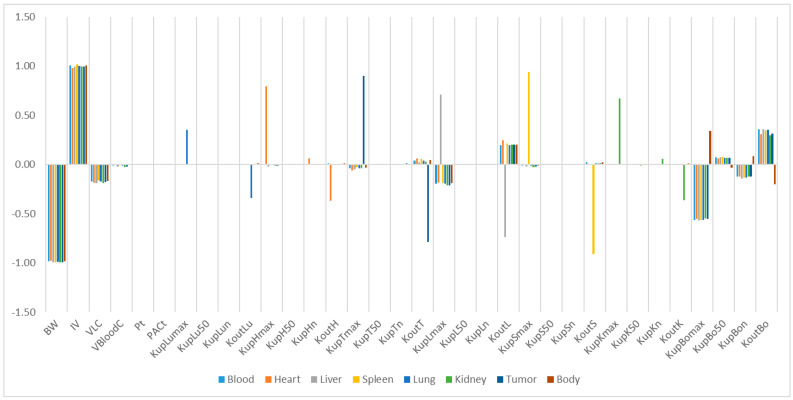
Relative sensitivity analyses for the parameters of mPEG5k-9.09%-80 nm NP concentrations in mouse tissues. Positive values represent PCL-P2 NP concentration increases in the organ when the parameter value increases, while negative values indicate PCL-P2 NP concentration decreases in the organ when the parameter value increases.

**Figure 8:**
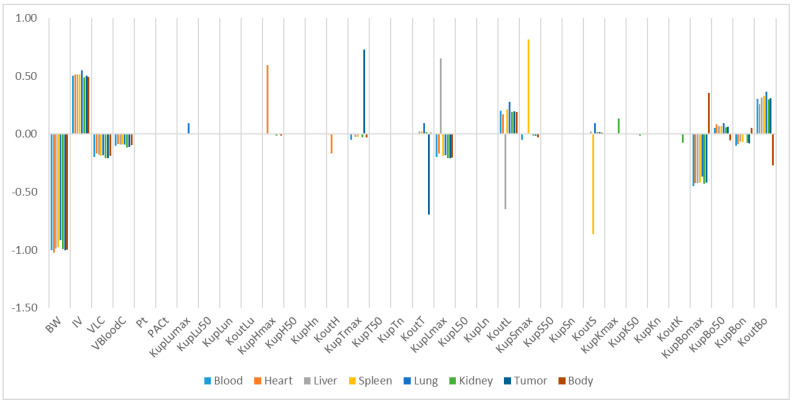
Relative sensitivity analyses for the parameters of mPEG5k-28.57%-80 nm NPs.

**Figure 9:**
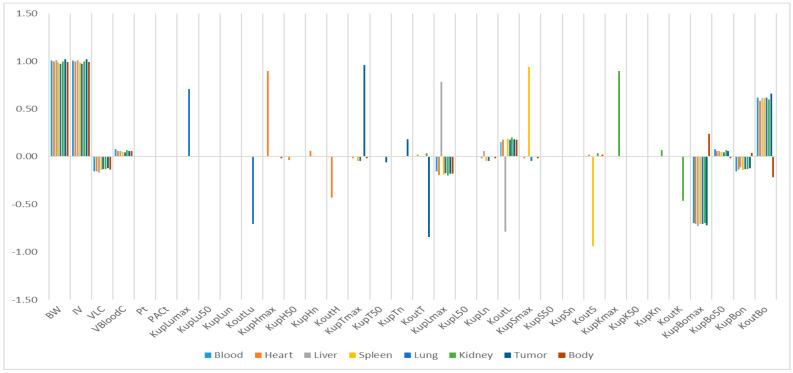
Relative sensitivity analyses for the parameters of mPEG5k-9.09%-200 nm NPs.

**Figure 10:**
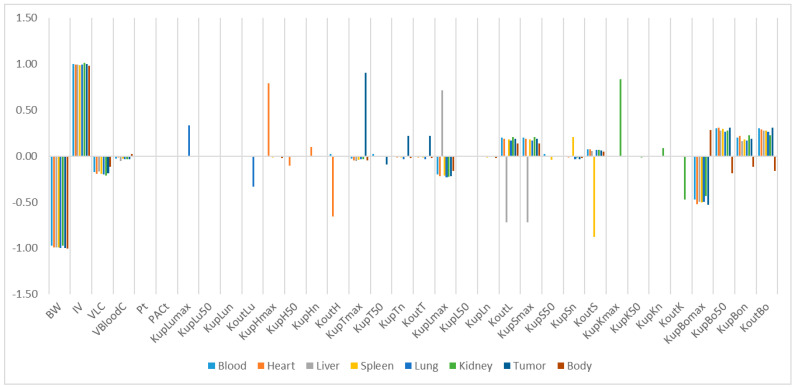
Relative sensitivity analyses for the parameters of mPEG5k-28.57%-200 nm NPs.

**Figure 11:**
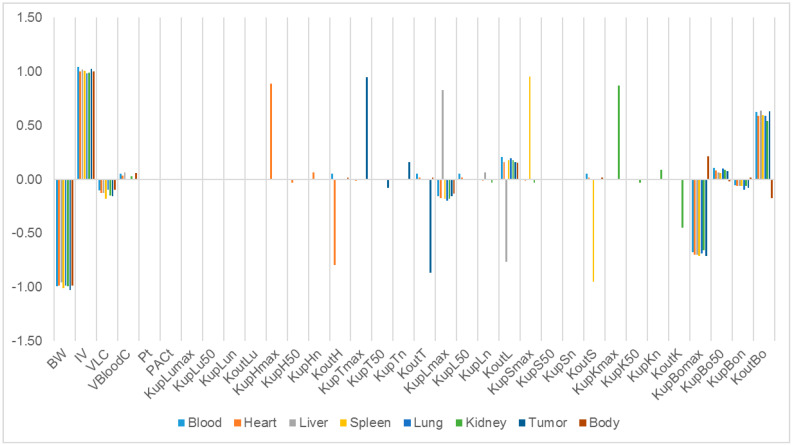
Relative sensitivity analyses for the parameters of mPEG2k-28.57%-200 nm NPs.

The relative sensitivity coefficients (RSC) related to physiological and NP-specific parameters for mPEG-PCL NP concentrations in bodily organs are shown in Tables S4–S8, and Figure 7, Figure 8, Figure 9, Figure 10 and Figure 11. Specifically, body weight (BW) and injected dose (IV) had a significant influence on all selected dose metrics regardless of the mPEG-PCL NP size and material of the study, while blood volume fraction (VBloodC) had minimal effects in terms of all dose metrics involving mPEG-PCL NPs. As for NP-specific parameters, the distribution coefficient (Pt) and permeability coefficient (PAC_t_) negligibly affected all mPEG-PCL NP concentrations in organs, which might indicate that the distribution of mPEG-PCL NPs was not determined by transcapillary transport. Among the phagocytosis-related parameters for each organ, mPEG-PCL NP concentration was highly sensitive to the maximum uptake (K_max_) and release rate constants (K_out_). As shown in Table S4 and Figure 7, relative sensitivity analyses for the parameters of mPEG5k-9.09%-80 nm NP concentrations in heart, liver, spleen, lung, kidney, and tumour were highly sensitive to K_up_H_max_, K_up_L_max_, K_up_S_max_, K_up_Lu_max_, K_up_K_max_,K_up_T_max_ and K_out_L, K_out_S, K_out_Lu, and K_out_T, respectively. K_up_50 and K_up_n determined the time and extent of activation of phagocytosis, and K_up_Bo_n_ had a significant influence on mPEG-PCL NP concentration in tissues. Similar effects of sensitivity analyses were observed for the other mPEG-PCL NPs, as shown in Tables S5–S8, and Figure 8, Figure 9, Figure 10 and Figure 11.

## 3. Discussion

It is important to understand the ADME properties of polymeric NPs because many of them are used in therapeutic and imaging fields [19,20]. PBPK modelling has been a useful tool for evaluating the in vivo distribution of NPs. However, to date, there are few papers that have referred to polymeric NPs in PBPK modelling [7,8,11,12,13,14,21,22], and so far, there have been no reports dealing with mPEG-PCL NPs using PBPK models. We synthesised five mPEG-PCL NP formulations involving different sizes, different ratios of mPEG-PCL/PCL, and different weights of coating mPEG. Then, a PBPK model for the five mPEG-PCL NP formulations was successfully built by directly fitting the observed concentration data at the organ level in mice. The PBPK modelling of five mPEG-PCL NPs can not only provide a strategy to study the in vivo behaviour of mPEG-PCL NPs, but also can help to extrapolate from animals to humans and predict likely pharmacokinetic parameters involving mPEG-PCL NPs in humans. In PBPK modelling of NPs, identifying integral particles from probe signals has been one of the biggest challenges in analytical approaches toward NPs [16]. It has been reported that the signals of 1, 10-dioctadecyl-3, 3, 30, 30-tetramethylindotricarbocyanine iodide dye-encapsulated NPs (DiR-NPs) can display sustained, high level fluorescence at even when DiR is released from NPs [23]. The radioactivity measurement of dissected animal organs is not only time consuming and tedious, but also it is difficult to determine whether the NPs are integrated particles [16]. In our studies, the water-quenching NIR fluorescent probe P2 has been proven one of the more accurate signals for monitoring the in vivo fate and integrity of NPs [23]. Thus, the PBPK model established in this study was much more accurate for predicting and simulating the in vivo biodistribution of mPEG-PCL NPs.

A very important issue for PBPK modelling of NPs is the selection of the model structure. According to transportation mechanisms, PBPK models are generally divided into two groups: blood-flow-limited and membrane-limited models [4]. Previous studies have shown that the pharmacokinetics of NPs with small sizes, such as PEG-coated gold NPs (AuNPs) of 13 nm, are well described by the membrane-limited model, whereas 100 nm AuNPs worked well in the blood-flow-limited model [7]. In the PBPK models of PEG/PAA NPs [11,12], blood flow- and membrane-limited models were adopted to describe the ADME process. The capillary blood and tissue compartments in the organs are described as membrane-limited processes. The two blood compartments were divided into two sub-compartments in which a blood-flow-limited process was adopted. In our PBPK models, we investigated both blood-flow- and membrane-limited models and found that membrane-limited models could be used to best simulate our experimental data. We also investigated whether phagocytosis in blood could help fit the model. However, no significant improvement was observed. Therefore, we did not include a subcompartment of PCs in the blood compartments. Thus, our PBPK structure was different to that of polymeric NPs and would be helpful for mPEG-PCL NPs in later PBPK modelling.

Some PBPK models of polymeric NPs did not consider the phagocytosis process [8,21], whereas a small number of studies do include them in PBPK models [7,11,12]. Phagocytosis is a mechanistic process by which specific cells engulf NPs, forming an internal compartment named the phagosome. These specific cells, namely PCs, include reticuloendothelial system cells (mononuclear phagocyte system cells), organ-located cells such as Kupffer cells in the liver, red pulp macrophages in the spleen, alveolar macrophages in the lungs, and intraglomerular mesangial cells in the kidney. It has been reported that NP uptake by PCs causes several side effects [2]. On the one hand, the body’s immune system can be damaged as NPs accumulate in organs over time. On the other hand, NPs resident in PCs could still act as a deposit inside the body and extend organ exposure duration when the stored NPs are slowly released from organs over time [11]. Thus, it is crucial to describe the contribution and uptake mechanism involving phagocytosis of PCs in the in vivo disposition of NPs for the development of their safe use. In our studies, mPEG-PCL NPs were taken up by PCs immediately after they were injected into the bloodstream, which was consistent with the results of previous studies regarding polymeric NPs using PBPK models [7,21]. Tumour compartments need to be carefully constructed owing to their abnormal physiological features as well as their different mechanisms and kinetics for the disposition of NPs in normal tissues [17]. To the best of our knowledge, we first attempted to integrate EPR effects into a tumour sub-compartment in a PBPK-based model involving polymeric NPs based on assumptions defined in previous studies [24]. The tumour in our PBPK model was considered as a compartment and divided into two sub-compartments, capillary blood and EPR, to study the EPR effects of mPEG-PCL NPs quantitatively (supporting information from the EPR-PBPK model). However, we found that it was still better to divide the tumour compartment into three sub-compartments, including PCs.

Our study identified some highly influential parameters in the biodistribution of mPEG-PCL NPs, such as BW, IV, K_max_, and K_out_ of the tissues. The sensitivity analysis showed that K_max_ and K_out_ were two of the most influential parameters determining mPEG-PCL NP concentrations in organs. This is consistent with previous research [7,11,20], indicating that phagocytosis is a key process for the biodistribution of mPEG-PCL NPs. We compared the K_max_ of the different NPs, which included phagocytosis as a compartment, and found that the K_max_ of PCs in our PBPK model was generally higher than those reported [7,11,20]. This might be the reason that polymeric NPs are capable of actively targeting organs and cells such as hepatocytes [21].

**Figure 12:**
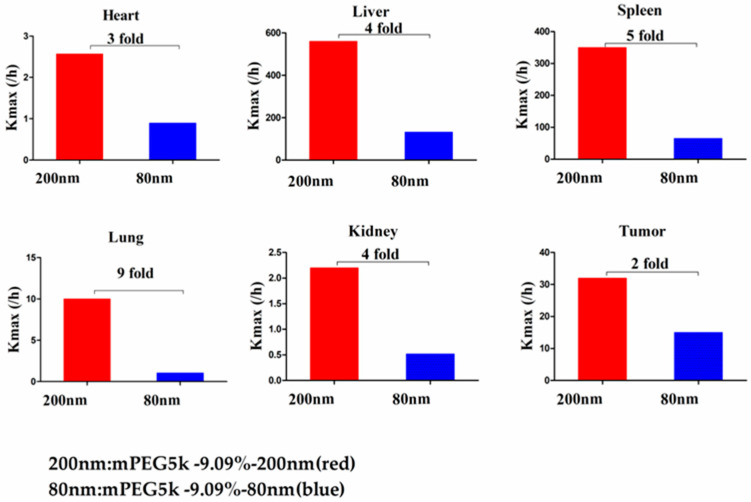
K_max_ (/h) for mPEG5k-9.09%-200 nm (red) and mPEG5k-9.09%-80 nm NPs (blue).

**Figure 13:**
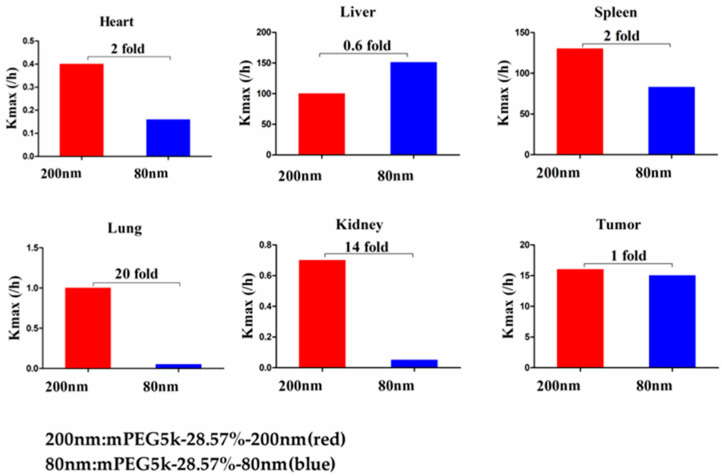
K_max_ (/h) for mPEG5k-28.57%-200 nm (red) and mPEG5k-28.57%-80 nm NPs (blue).

**Figure 14:**
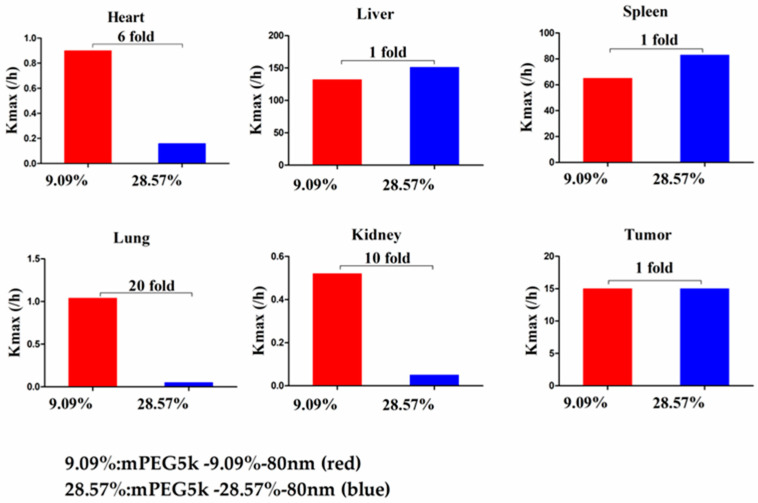
K_max_ (/h) for mPEG5k-9.09%-80 nm (red) and mPEG5k-28.57%-80 nm NPs (blue).

**Figure 15:**
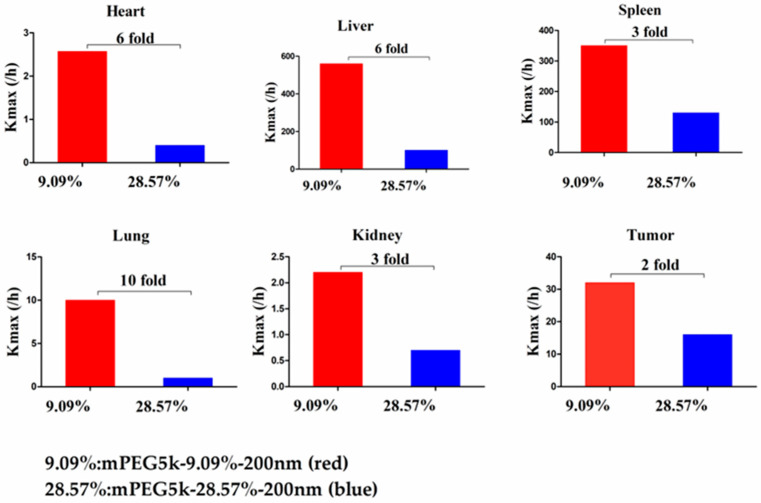
K_max_ (/h) for mPEG5k-9.09%-200 nm (red) and mPEG5k-28.57%-200 nm NPs (blue).

**Figure 16:**
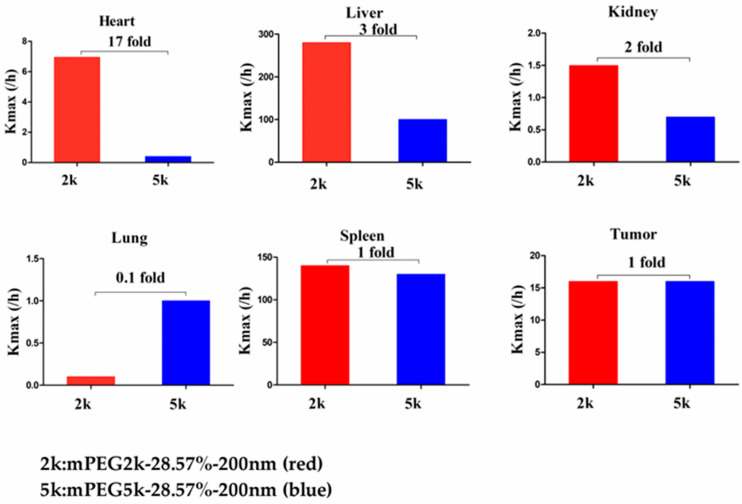
K_max_ (/h) for mPEG5k-28.57%-200 nm (red) and mPEG2k-28.57%-200 nm NPs (blue).

Compared with mPEG5k-9.09%-80 nm (Figure 12), K_max_ (/h) in the PBPK model for mPEG5k-9.09%-200 nm was > two times in each tissue, and the K_max_ (/h) in the lung was 9.62 times, and 2.13 times for tumours. Thus, we speculated that with the same mPEG5k modification of 9.09%, the 200 nm NPs were more easily phagocytosed by PCs in the tissues than 80 nm NPs, reducing the amount of NPs in blood, leading to a decreased amount circulating into the tumour tissue. For the two formulations of mPEG5k-28.57%-80 nm and mPEG5k-28.57%-200 nm (Figure 13), K_max_(/h), the PBPK models for liver and spleen tissues were both <two-fold different, indicating that in the same mPEG5k and 28.57% ratio modification, the diameter of NPs had no significant effect on the distribution of NPs in liver and spleen. However, for the heart, and especially for the lungs and kidneys, K_max_(/h) exhibited large differences, and the largest difference could reach 20-fold, indicating that NP size had a significant influence in these three tissues for the mPEG5k-28.57%-80 nm and mPEG5k-28.57%-200 nm formulations. There were almost no differences observed in tumours. From this, we speculated that with the 28.57% mPEG5k modification, the 200 nm NPs were more easily phagocytosed by the PCs in the heart, lung, and kidney, while the PCs in the liver, spleen, and in tumours were less affected. In the two formulations of mPEG5k-9.09%-80 nm and mPEG5k-28.57%-80 nm (Figure 14), the K_max_ (/h) in the PBPK model for liver and spleen tissues were both within two-fold. However, in the heart, and especially in the lung and kidney, the K_max_ (/h) difference was higher. The largest difference could reach 20-fold, and there was no difference in tumours. K_max_ (/h) in the PBPK model for mPEG5k-9.09%-200 nm was more than two-fold different in each tissue than mPEG5k-28.57%-200 nm (Figure 15). K_max_ (/h) in the lung was 10 times the maximum and two-fold higher in tumour tissue. It was concluded that NPs with less mPEG5k content are easily phagocytosed by PCs in tissues. In the two formulations of mPEG5k-28.57%-80 nm and mPEG2k-28.57%-200 nm (Figure 16), there was no significant difference for K_max_(/h) between the spleen and tumour tissues, indicating that under the same amount of 28.57% mPEG, the mPEG molecular weight had little effect on the distribution of NPs in spleen and tumour tissues. However, in the liver and kidneys, and especially in the heart, the K_max_ (/h) in the PBPK model was 17 times that of the two NP formulations. In the lungs, however, the opposite was observed. Therefore, we can speculate that in the two NP formulations with the same amount of 28.57% mPEG and 200 nm size, the mPEG molecular weight of 2K formulations was easily phagocytosed by the PCs of the heart, liver, and kidneys, and quite the opposite in the lungs, whereas no significant differences were observed for the spleen and tumours.

**Figure 17:**
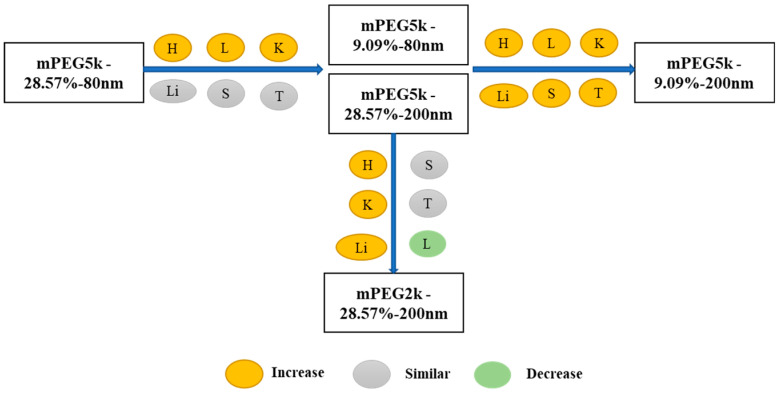
Multiple comparisons of K_max_ (/h) for the five NP formulations. The yellow circle indicates increase, grey means similar and green represents decrease in the K_max_ (/h) in various tissues.

In summary, as shown in Figure 17, compared with the mPEG5k-28.57%-80 nm formulation, the distribution of mPEG5k-9.09%-80 nm and mPEG5k-28.57%-200 nm in the heart, lung and kidney increased, reducing the circulating NP delivery to tumour sites. The distribution of mPEG5k-9.09%-200 nm was increased in all tissues. Compared with mPEG5k-28.57%-200 nm, mPEG2k-28.57%-200 nm levels were increased in the heart, liver, and kidney, but decreased in the lungs.

Pt is one of the key parameters in PBPK models that represent the tissue/plasma distribution coefficient. The value of Pt might be related to the formation of a biocorona complex around NPs because the composition of interstitial fluid differs from that of plasma [25]. Biocorona formation can not only dramatically alter the biodistribution of NPs, but also potentially impact interspecies extrapolation of NP biodistribution [24]. The formation of the biocorona can be influenced by the properties of the NPs, such as size, surface charge, and ligands, and then change the interactions involving NPs and the biological system [25]. In the PBPK model of PEG/PAA NPs [11], Pt was obtained by fitting the unknown model parameters to the experimental data. Here, we assigned the same Pt value from the published PBPK models for 100 nm Au NPs [7] for all organs except the liver. Similar to previously reported PBPK models of 100 nm Au NPs, the sensitivity analysis in our PBPK models showed that Pt had no significant effect on mPEG-PCL NP distribution, implying that greater changes to these parameters would have little influence on the model outputs. In contrast to the 13 nm Au NPs [7], Pt is a highly influential parameter on the model outputs. PAC_t_ is described as diffusion between capillary blood and tissue, which could affect how rapidly the NPs deviate from capillary blood into surrounding tissues. We used the same permeability coefficient for all organs except the spleen for our five types of mPEG-PCL NPs based on the reported PBPK models of 100 nm Au NPs [7]. According to the sensitivity analysis, PAC_t_ also had little influence on the output result in our PBPK model, which was consistent with the PBPK model of 100 nm Au NPs and in contrast to that of the PBPK model for 13 nm Au NPs. The permeability of NPs is regulated by opsonisation, in which proteins bind to NP surfaces almost instantaneously once NPs enter the blood circulation and form NP-protein complexes. Thus, the opsonisation process can change the physicochemical properties of the original NPs. In addition, the different morphological features of blood vessel endothelium, classified as continuous or discontinuous, also influence NP distribution from the blood compartment into tissues. Furthermore, blood supply to tissues may also play an important role in the permeability of NPs when they are distributed into the tissue very rapidly, or where the blood supply is quite limited in some tissues [11].

The extrapolation capacity of the PBPK model is highly desirable. Thus, external validation of the PBPK model is a crucial step in demonstrating the value of application of PBPK models. In the future, we will focus on these highly influential parameters to help understand the relationship between such properties and the biodistribution of mPEG-PCL NPs. In addition, more knowledge is needed regarding how mPEG-PCL NP properties influence PC uptake quantitatively in different organs in order to improve the predictive capacity of PBPK models.

## 4. Materials and Methods

### 4.1. Chemicals

Poly(ε-caprolactone) (PCL, MW = 45,000) and polyvinyl alcohol (PVA, MW = 13,000–23,000, 87–89% alcoholised) were supplied by Sigma–Aldrich (St. Louis, MO, USA). Methoxy PEG-poly(ε-caprolactone) (mPEG-PCL) copolymers with different mPEG and PCL chain lengths (mPEG_5k_-PCL_45k_, mPEG_2k_-PCL_45k_) were synthesised according to previous procedures [26,27] and were kindly provided by Professor Zhiyong Qian of Sichuan University, Chengdu, China. The fluorescent probe P2 (*λ*_abs_/*λ*_em_ = 708/732 nm) was synthesised in our lab according to previous procedures [28,29]. Dichloromethane (DCM was purchased from Sinopharm Chemical Reagent Co., Ltd. (Shanghai, China). Roswell Park Memorial Institute (RPMI)-1640, Dulbecco’s modified Eagle’s medium (DMEM), penicillin streptomycin (PenStrep), 0.25% trypsin–0.02% ethylenediamine tetraacetic acid (EDTA), and D-Hank’s balanced salt solution were purchased from Gibco (USA). Foetal bovine serum (FBS) was supplied by Invitrogen (Carlsbad, CA, USA). Purified water was prepared using a Milli-Q purification system (Molsheim, France). All other reagents used in this study were of analytical grade and used as received.

### 4.2. Preparation of Fluorescently Labelled mPEG-PCL NPs

mPEG-PCL NPs labelled with P2 were prepared by an oil-in-water type emulsification/solvent evaporation method according to our previous work [5]. Briefly, as shown in Table S1, 200 mg of PCL and a certain amount of mPEG-PCL, together with the fluorescent probe (100 μg), were dissolved in 5 mL of DCM to form an organic phase. The aqueous phase was 20 mL of a 1% (*w/w*) PVA solution. The organic phase was then mixed with the aqueous phase and emulsified by probe ultrasonication (Scientz Biotechnology Co., Ltd., Ningbo, China) for 3 min in an ice bath to obtain a coarse emulsion. After mechanical stirring for 4 h at room temperature to remove the DCM, mPEG-PCL NPs with a particle size of 200 nm were obtained. The 80-nm NPs were prepared using 20 mL of 5% (*w/w*) PVA solution as the aqueous phase, subsequently disrupting the coarse emulsion at a pressure of 1000 bar for 3 min using a high-pressure homogeniser (Scientz Biotechnology Co.), followed by mechanical stirring for 4 h to remove the residual DCM. The obtained NP suspensions were stored at 4 °C for further analysis and animal experiments. NPs with different PEG coating chain lengths and coating densities were prepared by incorporating various amounts of mPEG-PCL with different mPEG chain lengths into the PCL matrix.

### 4.3. Characterisation of mPEG-PCL NPs

The physicochemical properties of various NPs were characterised after 20-fold dilution with purified water. The hydrodynamic diameter and zeta potential were determined using a Zetasizer Nano (Malvern Instruments, Malvern, Liverpool, UK) at 25 °C. The particle size was reported as the Z-average value. Samples (100 μL) were added to a 96-well plate and fluorescently quantified using an IVIS^®^spectrum live imaging system (PerkinElmer, CA, USA) with excitation/emission wavelengths of 710/760 nm.

### 4.4. Cell Culture

The murine carcinoma cell line S180, originating from the American Type Culture Collection (ATCC), was cultured as previously reported [30]. Briefly, S180 cells were seeded and cultured at 37 °C, 5% CO_2_, and 90% relative humidity in a 25 cm^2^ culture flask. The culture medium, composed of RPMI 1640 with 10% FBS, 1.0 mM sodium pyruvate, 0.1 mM nonessential amino acids and 1.5 g/L sodium bicarbonate, was changed every second day.

### 4.5. Biodistribution of mPEG-PCL NPs in S180-Bearing Mice

Kunming mice (18 ± 2 g) were subcutaneously inoculated with S180 tumour cells (1–2 × 10^6^ cells per mouse) via the right hindlimb, and subsequently raised under 25 ± 2 °C, 75 ± 5% relative humidity, and natural daylight for 8–12 days. Tumour diameters were measured every other day, and tumour volumes were calculated as (a^2^ × b)/2, where **a** and **b** refer to the long and short diameters, respectively. Animals with similar tumour volumes (0.51–0.59 cm^3^) were included for further study.

The tumour-bearing mice were injected intravenously with 0.2 mL of mPEG-PCL NP suspension. All formulations were adjusted to the same fluorescent intensity level by slight dilution with deionised water. The biodistribution of mPEG-PCL NPs in S180 tumour-bearing mice was first investigated by whole animal imaging using an IVIS spectrum live imaging system with excitation/emission wavelengths of 710/760 nm. After administration, images of mice were captured at predetermined time intervals for as long as 72 h, whereas blank images were taken before administration as a control. During the image-capturing process, animals were narcotised using an on-line gas anaesthesia system using isoflurane (Shandong Keyuan Pharmaceutical Co., Ltd., China).

To further investigate the biodistribution of mPEG-PCL NPs in tumour-bearing mice, the animals were sacrificed and dissected at predetermined time intervals of 0.083, 1, 4, 8, 24, and 72 h post administration. Various organs and tissues were collected after cardiac perfusion and imaged using an IVIS live imaging system, as mentioned above, to visualise the mPEG-PCL NPs. Blood samples were withdrawn prior to perfusion for further quantification.

To accurately quantify mPEG-PCL NPs in organs and tissues, samples were cut into pieces and homogenised using a high-shear homogeniser (IKA, CA, USA). Water was excluded during homogenisation to remove any potential influence on the quenching of the P2 probe encapsulated in the NPs. Then, 100 μg of each sample was precisely weighed and added to a 96-well plate. The fluorescence of the samples was measured using an IVIS spectrum live imaging system with excitation/emission wavelengths of 710/760 nm. Images were captured using an automatic exposure mode, regions of interest (ROIs) were drawn over the fluorescent signals, and the total radiant efficiency (TRE) within the ROIs was measured by vendor software for subsequent quantitative analysis [18,23]. The fluorescence of the blood samples was measured as mentioned above, whereas homogenisation was excluded. All quantification of NPs was conducted based on the corresponding calibration curve, which was made by plotting the fluorescence of standard samples versus the concentration of mPEG-PCL NPs. Standard samples were prepared by adding a specific amount of NPs to organs or tissues, followed by homogenisation, as described above. The linear ranges of calibration curves for all formulations were 0.0078125–0.5 mg/g for the heart, 0.0078125–1 mg/g for the liver, 0.0078125–1 mg/g for the spleen, 0.00390625–0.5 mg/g for the lung, 0.00390625–0.5 mg/g for the kidney, 0.00390625–1 mg/g for tumours, and 0.01–0.5 mg/g for blood. The linear equation and corresponding linear coefficients are shown in Table S2.

### 4.6. PBPK Modelling

**Figure 18:**
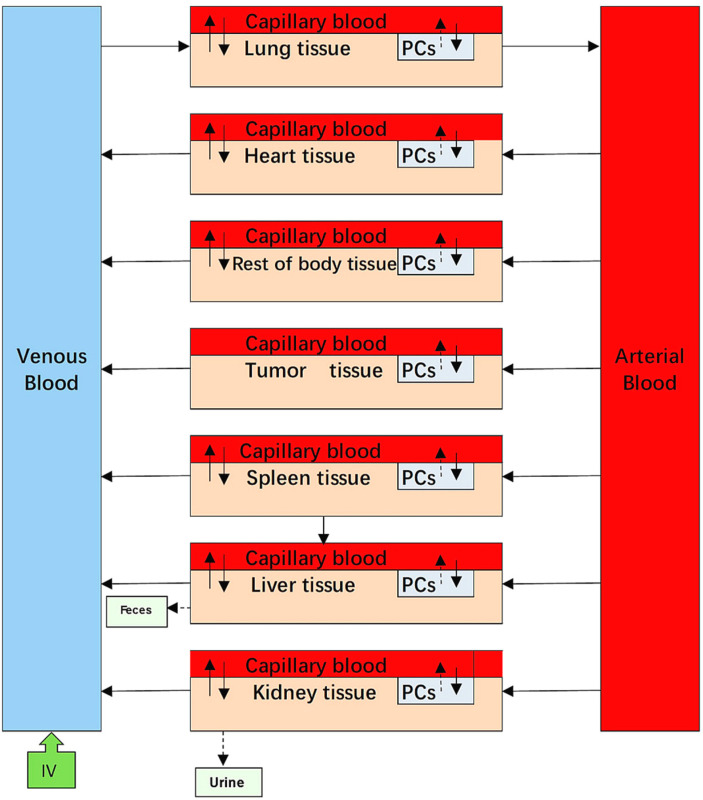
Structure of a blood flow-limited PBPK model for all the five mPEG-PCL NPs formulations, including phagocytosing sub-compartment in organs shortened as “PCs.” Arrows show the transportation of mPEG-PCL NPs while grey boxes indicate the uptake of mPEG-PCL NPs by PCs separated from blood circulation or tissues. Here the tissue represents interstitial space of organs when used to simulate our experimental data of mPEG-PCL NPs.

The PBPK model was divided into nine compartments: venous blood, arterial blood, heart, liver, spleen, lungs, kidneys, and tumour, which are regarded as physiological organ compartments, as well as the rest of the body. Each organ compartment consisted of three sub-compartments: capillary blood, tissue, and PCs. All compartments were interconnected by venous and arterial blood. According to the available literature, NPs can be taken up via a wide variety of endocytic pathways [16]. To simplify these complex pathways, two general pathways were generated based on the transportation mechanisms involved. In the blood-flow-limited method, NPs were directly transferred from the bloodstream into macrophages located at the tissue capillary blood vessels, while the membrane-limited method was that the NPs were diffused into tissue and further taken up by tissue macrophages. Based on the earlier approaches employed in the reported NP PBPK models [7,11], we attempted to describe phagocytosis of 80 nm and 200 nm mPEG-PCL NPs by both blood flow- and membrane-limited processes. We found that it was better to describe phagocytosis using a flow-limited method for all five mPEG-PCL NP formulations (Figure 18).

Based on mass balances, we used three main model equations to describe the extent of cellular uptake of mPEG-PCL NPs from the blood (or interstitial space of tissue) and their subsequent release back to the blood (or interstitial tissue spaces) in the three sub-compartments. The mathematical equations that explain these processes are as follows:

For blood space:

(1) $$V_{V\_t}\frac{dCV_{t}}{dt}=Q_{T}\left(C_{A}-CV_{t}\right)-PA_{t}CV_{t}+\frac{PA_{t}C_{T\_t}}{P_{t}}-k_{up_{\_t}}CV_{t}V_{V\_t}+k_{release\_t}A_{PC\_t}$$

For tissue space:

(2) $$V_{T\_t}\frac{dC_{T\_t}}{dt}=PA_{t}CV_{t}-\frac{PA_{t}C_{T\_t}}{P_{t}}$$

For PCs in organs:

(3) $$\frac{dA_{PC\_t}}{dt}=k_{up_{\_t}}CV_{t}V_{V\_t}-k_{release\_t}A_{PC\_t}$$

where *P_t_* represents the tissue/plasma distribution coefficient for organ t; *PA_t_* represents the permeability area cross product between the blood and the tissue of the organ t (*PA_t_* = *PAC_t_* × *Q_t_*; *PAC_t_* represents the permeability coefficient of NPs between capillary blood and tissue); *CV_t_* represents the mPEG-PCL NP concentration in the venous blood of the organ t, *A_PC_t_* represents the amount of mPEG-PCL NPs in the PC sub-compartment of organ t, *Q_t_* represents the blood flow to organ t, *V_V_t_* represents the blood volume of the organ t, *V_T_* represents tissue volume (interstitial space for mPEG-PCL NPs), CA represents the mPEG-PCL NP concentration in the arterial blood, *C_T_t_* is the MPEG-PCL NP concentration in the tissue sub-compartment of the organ t, *k_up_t_* is the uptake rate constant of organ t, and *K_out___t_* is the release rate constant of organ t. More detailed information related to the process of the model and the mathematical equations are provided in the Supporting Information (physiological parameters used in the PBPK model for NPs in mice Table S3; mass balance equations).

It was suggested that the processes of release and excretion of NPs in organs were assumed to follow first-order kinetics. Because the uptake rate of PCs decreases as the amount of captured NPs approaches PC saturation levels, the time-dependent uptake rate constant (*k_up_*) of mPEG-PCL NPs by PCs can be accurately described by the Hill function, as based on previous reports [5,7].

(4) $$K_{up_{\_t}}=\frac{K_{\max\_t}*T^{n_{t}}}{K_{50\_t}^{n_{t}}+T^{n_{t}}}$$

where *K*_max_ is the maximum uptake rate constant, *K*_50_ is the time required to reach half of *K*_max_, *T* is the time, and *n* is the Hill coefficient.

### 4.7. EPR-PBPK Model for mPEG-PCL NPs

**Figure 19:**
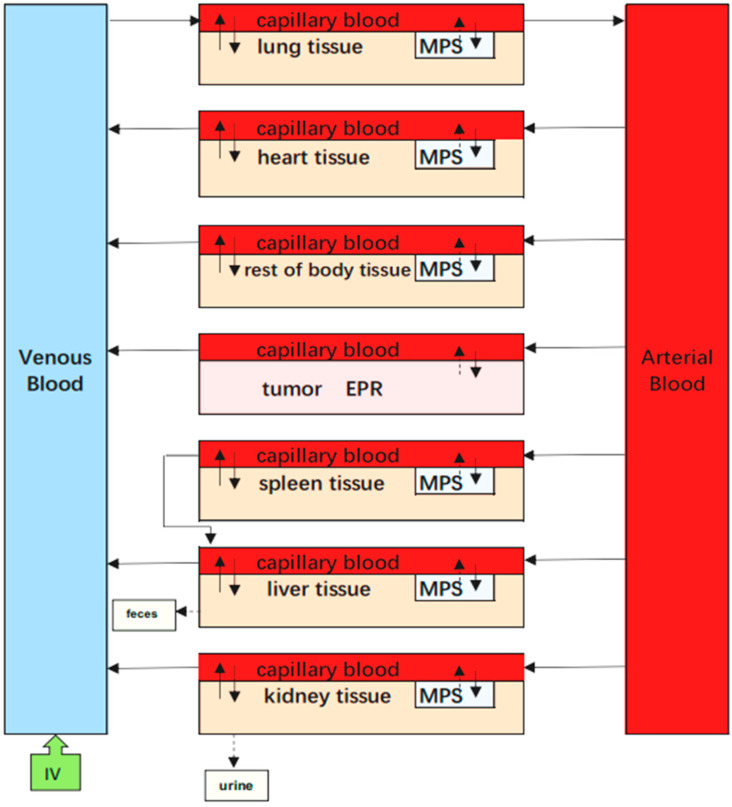
Schematic diagrams illustrating the PBPK model for mPEG-PCL NPs, including mononuclear phagocytic system in organs represented as “MPS” and enhanced permeability and retention in tumours represented as “EPR.” Arrows indicate the direction of transport of mPEG-PCL NPs, while grey boxes indicate the uptake of mPEG-PCL NPs by MPS or EPR from blood circulation or tissues.

In addition to the PBPK model describing the dynamics of mPEG-PCL NPs between and within organs involved in cellular phagocytosis, we also tried to integrate EPR effects as a sub-compartment to evaluate the EPR effects on tumours (Figure 19).

In this PBPK model, the tumour was divided into two compartments, namely the vascular and EPR spaces. Here, we considered the EPR as an independent compartment, which was described by first-order rate constants of *K*_1_ and *K*_2_ to quantitatively investigate the transfer of NPs between the vascular and EPR spaces in tumour tissue.

For vascular space:

(5) $$V_{V\_T}\frac{dCV_{T}}{dt}=Q_{T}\left(\mathrm{CA}-CV_{T}\right)-K_{1}CV_{T}V_{V_{T}}+K_{2}A_{EPR\_T}$$

For EPR space in tumour:

(6) $$\frac{dA_{PC\_T}}{dt}=K_{1}CV_{T}V_{V\_T}-K_{2}A_{EPR\_T}$$

Parameters such as *V_V_T_*, *CV_T_*, $CV_{T},\;V_{V_{T}}$ could be collected from the literature. The rate constant *K*_1_ represents the extravasation of mPEG-PCL NPs from circulation into the tumour, while the rate constant *K*_2_ describes the intravasation of mPEG-PCL NPs from the tumour back into the circulation. $A_{EPR\_T}$ represents the mPEG-PCL NP concentration in the tumour-EPR compartment.

We used Berkeley Madonna v.8.3.23 (University of California, Berkeley, CA, USA) to execute the code (the code in the PBPK model for IV injection of NPs can be found in the Supporting Information) for the PBPK model, and to run the simulations. The initial values of P and PAC were obtained from the PBPK model for Au NPs [7] and were further optimised by visually fitting to the measured data of mPEG-PCL NP concentrations after intravenous injection. The mPEG-PCL NP-specific parameters (*K*_max_, *K*_50_, *K*_out_, and *n*) for each organ were estimated in a tissue-by-tissue manner by fitting the concentration–time of mPEG-PCL NPs in both tissue and plasma samples into a simplified “open loop” model with only one tissue compartment. Curve fitting performed with Berkeley Madonna was conducted to obtain the minimal weighted least squares fit of the model between the predicted and experimental values. The values of the parameters obtained from the “open loop” models were then used as initial estimates in the whole-body PK model [5]. The concentration–time profiles of mPEG-PCL NPs in tissues and plasma were fitted simultaneously to obtain mPEG-PCL NPs and tissue-specific parameters.

### 4.8. Goodness of Fit

First, we visually compared the time courses involving the amounts of NPs between the experimental data and predicted concentrations in the blood and in various tissues in both the PCs-PBPK and EPR-PBPK models. Then, linear regression analyses of log values for all the experimental and predicted concentrations were conducted, and R^2^ values were compared to assess the goodness of fit (Figures S3 and S9).

### 4.9. Sensitivity Analysis

To determine the most influential parameters in the model simulation, sensitivity analysis was performed for all organs until 24 h after injection. First, we separately multiplied and divided each parameter by a factor of 2 and compared the NP amount in a given organ such as the liver compartment over time to determine the relative sensitivity parameters. Then, the values of relative sensitivity parameters were increased by 1% and the area under the curve (AUC) for the mPEG-PCL NP concentrations was analysed from the model simulations in all the compartments except for blood. The relative sensitivity coefficients (RSC) were calculated for all parameters (p) and compartments, as shown in Equation (5). If the absolute values of RSC were >0.5, they were viewed as highly sensitive parameters.

(7) $$\mathrm{RSC}=\frac{dAUC/AUC}{dP/P}$$

## 5. Conclusions

In this study, a PC-PBPK model was developed to mathematically describe the dynamics of mPEG-PCL NPs within and between organs in mice. The PC-PBPK model can be used to quantitatively describe and predict concentration–time profiles and exposure of the five NP formulations in blood and various organs. The sensitivity analysis showed that K_max_ and K_out_ were two of the most influential parameters in determining mPEG-PCL NP concentrations in organs; this is the first study using PBPK modelling to simulate the in vivo fate of polymeric NPs traced by environmentally responsive near-infrared dye. It is beneficial to understand the in-depth mechanism of polymeric NPs in vivo. By considering interspecies differences in physiological- and chemical-specific parameters, the PBPK model could also be extrapolated to rats and humans.

## Figures and Tables

**Table 1:** Goodness of fit for physiologically based pharmacokinetic (PBPK) model of NP formulations (R^2^). PCs: phagocytic cells; EPR: enhanced permeability and retention.

Formulations	PCs-PBPK Model	EPR-PBPK Model
mPEG5k-9.09%-80 nm	0.8810	0.8420
mPEG5k-28.57%-80 nm	0.9068	0.7567
mPEG5k-9.09%-200 nm	0.7755	0.8290
mPEG5k-28.57%-200 nm	0.8091	0.7033
mPEG2k-28.57%-200 nm	0.8561	0.7725

**Table 2:** mPEG-PCL NP-specific descriptions and values of parameters in the PCs-PBPK model.

Parameters (Unit)	Description	Size and Material	Lung	Liver	Spleen	Kidney	Tumour	Heart	Rest of Body
P (unitless)	tissue/plasma distribution coefficient	mPEG5k-9.09%-80 nm	0.15	0.08	0.15	0.15	0.15	0.15	0.15
		mPEG5k-28.57%-80 nm	0.15	0.08	0.15	0.15	0.15	0.15	0.15
		mPEG5k-9.09%-200 nm	0.15	0.08	0.15	0.15	0.15	0.15	0.15
		mPEG5k-28.57%-200 nm	0.15	0.08	0.15	0.15	0.15	0.15	0.15
		mPEG2k-28.57%-200 nm	0.15	0.08	0.15	0.15	0.15	0.15	0.15
PAC (unitless)	permeability coefficient	mPEG5k-9.09%-80 nm	0.001	0.001	0.03	0.001	0.001	0.001	0.001
		mPEG5k-28.57%-80 nm	0.001	0.001	0.03	0.001	0.001	0.001	0.001
		mPEG5k-9.09%-200 nm	0.001	0.001	0.03	0.001	0.001	0.001	0.001
		mPEG5k-28.57%-200 nm	0.001	0.001	0.03	0.001	0.001	0.001	0.001
		mPEG2k-28.57%-200 nm	0.001	0.001	0.03	0.001	0.001	0.001	0.001
K_max_ (h^−1^)	maximum uptake rate constant	mPEG5k-9.09%-80 nm	1.04	132	65	0.52	15	0.9	4
		mPEG5k-28.57%-80 nm	0.05	151	83	0.05	15	0.16	2
		mPEG5k-9.09%-200 nm	10	560	350	2.2	32	2.57	51
		mPEG5k-28.57%-200 nm	1	100	130	0.7	16	0.40	21
		mPEG2k-28.57%-200 nm	0.1	280	140	1.5	16	6.94	28
K_50_ (h)	time for reaching half-maximum uptake rate	mPEG5k-9.09%-80 nm	0.006	0.001	0.003	0.04	0.02	0.4	0.5
		mPEG5k-28.57%-80 nm	0.006	0.001	0.003	0.04	0.02	0.4	1
		mPEG5k-9.09%-200 nm	0.0052	0.0008	0.003	0.04	0.3	0.4	0.09
		mPEG5k-28.57%-200 nm	0.0002	0.46	0.0056	0.012	0.3	0.4	10
		mPEG2k-28.57%-200 nm	0.0002	0.04	0.01	0.012	0.3	0.4	0.4
n (unitless)	Hill coefficient	mPEG5k-9.09%-80 nm	0.001	0.001	1.62	0.04	0.005	0.07	0.5
		mPEG5k-28.57%-80 nm	0.001	0.001	1.6	0.04	0.005	0.07	1
		mPEG5k-9.09%-200 nm	0.002	0.02	1.6	0.04	0.2	0.07	0.5
		mPEG5k-28.57%-200 nm	0.0002	0.5	0.4	0.04	0.2	0.07	1
		mPEG2k-28.57%-200 nm	0.0002	0.9	0.01	0.04	0.2	0.07	1
K_out_ (h^−1^)	release rate constant	mPEG5k-9.09%-80 nm	0.92	5	3	0.085	0.27	0.075	0.10
		mPEG5k-28.57%-80 nm	4	12	7.2	0.085	1.1	0.075	0.17
		mPEG5k-9.09%-200 nm	2	7	3.6	0.085	0.53	0.075	0.41
		mPEG5k-28.57%-200 nm	1	9	1.6	0.085	0.53	0.075	0.94
		mPEG2k-28.57%-200 nm	1	11	1.4	0.085	0.53	0.4	0.32
Kbile or Kurine (L/h)	biliary or urinary clearance	mPEG5k-9.09%-80 nm	NA	0.0012	NA	0.00012	NA	NA	NA
		mPEG5k-28.57%-80 nm	NA	0.0012	NA	0.00012	NA	NA	NA
		mPEG5k-9.09%-200 nm	NA	0.0012	NA	0.00012	NA	NA	NA
		mPEG5k-28.57%-200 nm	NA	0.0012	NA	0.00012	NA	NA	NA
		mPEG2k-28.57%-200 nm	NA	0.0012	NA	0.00012	NA	NA	NA

## Data Availability

Data of the compounds are available from the authors.

## Supplementary Materials

The following are available online, Figure S1: Size distributions of various mPEG-PCL nanoparticles encapsulating P2, Figure S2: Whole animal imaging of S180 tumor bearing mice after intravenous administration of mPEG-PCL nanoparticles at the leteral position (A–E) and supine position (F–J), Figure S3: Goodness-of-fit plot of the linear regression analysis of model predictions and experimental data after intravenous injection for model calibration. Measured values are from our own experimental data. mPEG5k-9.09%-80 nm NPs (A), mPEG5k-28.57%-80 nm NPs (B), mPEG5k-9.09%-200 nm NPs (C), mPEG5k-28.57%-200 nm NPs (D) and mPEG2k-28.57%-200 nm NPs (E), Figure S4: The simulated and experimentally measured concentration of mPEG5k-9.09%-80 nm NPs in mice blood and tissues after intravenous injection. Simulation results are represented by solid lines in each panel, and the mean values of measured data are represented by red circles. Error bars represent standard deviation of experimentally measured data, Figure S5: The simulated and experimentally measured concentration of mPEG5k-28.57%-80 nm NPs in mice blood and tissues after intravenous injection, Figure S6: The simulated and experimentally measured concentration of mPEG5k-9.09%-200 nm NPs in mice blood and tissues after intravenous injection, Figure S7: The simulated and experimentally measured concentration of mPEG5k-28.57%-200 nm NPs in mice blood and tissues after intravenous injection, Figure S8: The simulated and experimentally measured concentration of mPEG2k -28.57%-200 nm NPs in mice blood and tissues after intravenous injection, Figure S9: Goodness-of-fit plot of the linear regression analysis of model predictions and experimental data after intravenous injection for model calibration. Measured values are from our own experimental data. mPEG5k-9.09%-80 nm NPs (A), mPEG5k-28.57%-80 nm NPs (B), mPEG5k-9.09%-200 nm NPs (C), mPEG5k-28.57%-200 nm NPs (D) and mPEG2k-28.57%-200 nm NPs (E), Table S1: Compositions and physicochemical properties of mPEG-PCL nanoparticles, Table S2: Linear relationship between fluorescence and concentrations of nanoparticles in various organs and tissues, Table S3: Physiological parameters used in the PBPK model for NPs in mice, Table S4: Relative sensitive analyses for the parameters of mPEG5k-9.09%-80 nm NPs, Table S5: Relative sensitive analyses for the parameters of mPEG5k-28.57%-80 nm NPs Table S6: Relative sensitive analyses for the parameters of mPEG5k-9.09%-200 nm NPs, Table S7: Relative sensitive analyses for the parameters of mPEG5k-28.57%-200 nm NPs, Table S8: Relative sensitive analyses for the parameters of mPEG5k-28.57%-200 nm NPs.

## Sample Availability

Samples of the compounds are available from the authors.
