# Supplementary material for: Simulation of the In Vivo Fate of Polymeric Nanoparticles Traced by Environment-Responsive Near-Infrared Dye: A Physiologically Based Pharmacokinetic Modelling Approach
Source: Molecules. 2021 Feb 26;26(5):1271. doi: 10.3390/molecules26051271 (PMC7956253; doi:10.3390/molecules26051271)
Supplement: Supplementary file 1 [file molecules-26-01271-s001.pdf]

Simulation of the *in Vivo* Fate of Polymeric Nanoparticles Traced by Environment-Responsive Near-Infrared Dye: A Physiologically Based Pharmacokinetic Modelling Approach

Table S1. Compositions and physicochemical properties of mPEG-PCL nanoparticles

	Nanoparticle formulation code				
	$mPEG_{5k}$-28.57%-200 nm	$mPEG_{5k}$-28.57-80 nm	$mPEG_{5k}$-9.09%-200 nm	$mPEG_{5k}$-9.09%-80 nm	$mPEG_{2k}$-28.57%-200 nm
$mPEG_{5k}$-PCL_{45k} (mg)	80	80	20	20	/
$mPEG_{2k}$-PCL_{45k} (mg)	/	/	/	/	40
PCL (mg)	200	200	200	200	200
P2 (μg)	100	100	100	100	100
mPEG-PCL content (%)	28.57	28.57	9.09	9.09	28.57
mPEG-PCL/PCL	4/10	4/10	1/10	1/10	4/10
Particle size (nm)[a]	213.70±3.32	84.89±2.85	201.87±6.64	77.48±3.91	207.60±3.28
PDI[a]	0.03	0.15	0.05	0.12	0.06
Zeta potential (mV)[a]	-3.59±0.39	-3.44±0.27	-2.80±0.23	-2.403±0.31	-4.11±0.12

[a] Measured at 25 °C after dilution with purified water by 20 folds. All measurements were conducted in triplicate.

Table S2. Linear relationship between fluorescence and concentrations of nanoparticles in various organs and tissues

	$mPEG_{5k}$-28.57%-80nm	$mPEG_{5k}$-9.09%-80nm	$mPEG_{5k}$-28.57%-200nm	$mPEG_{5k}$-9.09%-200nm	$mPEG_{2k}$-28.57%-200nm
heart	$Y=4.207*10^8C+9.055*10^5$	$Y=4.043*10^8C+8.828*10^5$	$Y=7.070*10^8C+6.549*10^5$	$Y=6.676*10^8C+7.421*10^5$	$Y=6.721*10^8C+7.314*10^5$
	$R^2 = 0.9755$	$R^2 = 0.9756$	$R^2 = 0.9751$	$R^2 = 0.9757$	$R^2 = 0.9817$
liver	Y=9.995*108C-1.344*107	$Y=1.006*10^9+1.422*10^6$	$Y=2.071*10^9C+1.981*10^6$	$Y=2.228*10^9C-1.625*10^7$	$Y=1.955*10^9C-1.281*10^6$
	$R^2 = 0.9963$	$R^2 = 0.9992$	$R^2 = 0.9982$	$R^2 = 0.9982$	$R^2 = 0.9876$
spleen	$Y=4.849*10^8C-1.520*10^7$	$Y=4.911*10^8C-4.324*10^6$	$Y=9.223*10^8C+1.472*10^7$	$Y=9.420*10^8C-3.449*10^6$	$Y=9.085*10^8C+2.192*10^6$
	$R^2 = 0.9865$	$R^2 = 0.9835$	$R^2 = 0.9995$	$R^2 = 0.9938$	$R^2 = 0.9925$
lung	$Y=1.899*10^9C+7.940*10^6$	$Y=2.215*10^9C+1.266*10^7$	$Y=3.606*10^9C-3.716*10^6$	$Y=3.366*10^9C+1.197*10^7$	$Y=3.868*10^9C+4.425*10^7$
	$R^2 = 0.9941$	$R^2 = 0.9979$	$R^2 =0.9938$	$R^2 = 0.9984$	$R^2 = 0.9960$
kidney	$Y=8.513*10^8C+6.180*10^6$	$Y=9.300*10^8C+1.032*10^7$	$Y=1.543*10^9C+5.304*10^6$	$Y=1.742*10^9C+1.338*10^6$	$Y=1.591*10^9C+8.401*10^5$
	$R^2 = 0.9956$	$R^2 = 0.9997$	$R^2 = 0.9971$	$R^2 = 0.9979$	$R^2 = 0.9908$
tumor	$Y=2.084*10^9C+8.987*10^5$	$Y=1.945*10^9C-7.943*10^6$	$Y=3.891*10^9C-6.382*10^6$	$Y=3.559*10^9C-6.632*10^5$	$Y=3.449*10^9C+9.662*10^6$
	$R^2 = 0.9983$	$R^2 = 0.9978$	$R^2 = 0.9979$	$R^2 = 0.9991$	$R^2 = 0.9994$
blood	$Y=8.899*10^8C-4.916*10^7$	$Y=9.170*10^8C-8.731*10^6$	$Y=1.609*10^9C-1.578*10^5$	$Y=1.959*10^9C+1.488*10^7$	$Y=2.106*10^9C-8.926*10^6$
	$R^2 = 0.9988$	$R^2 = 0.9985$	$R^2 = 0.9999$	$R^2 = 0.9998$	$R^2 = 0.9992$

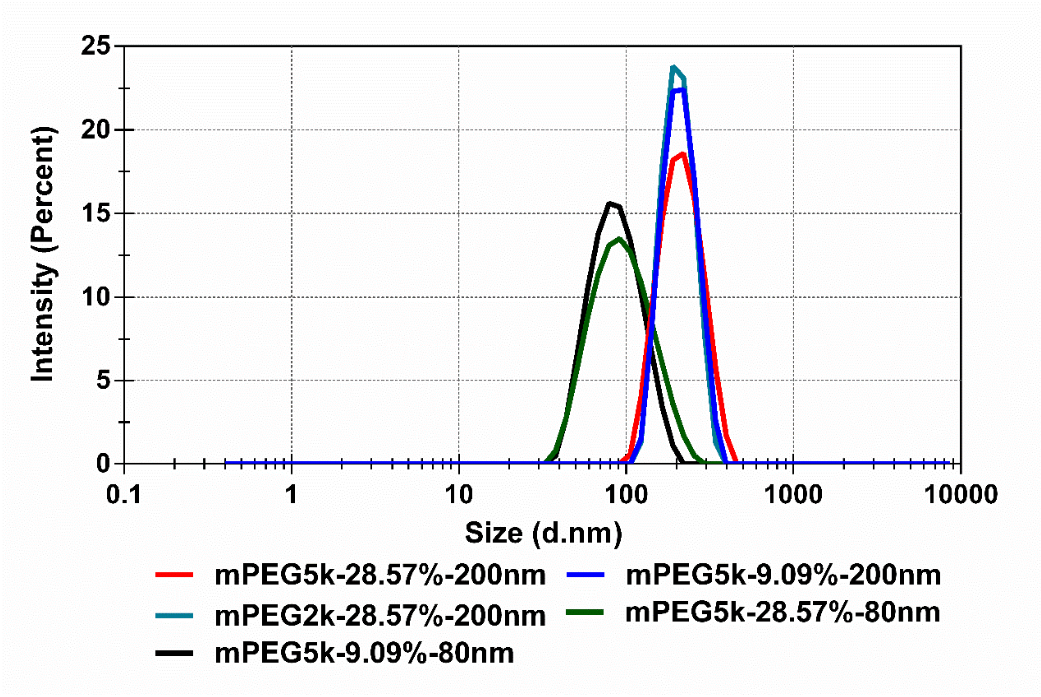

Figure S1. Size distributions of various mPEG-PCL nanoparticles encapsulating P2.

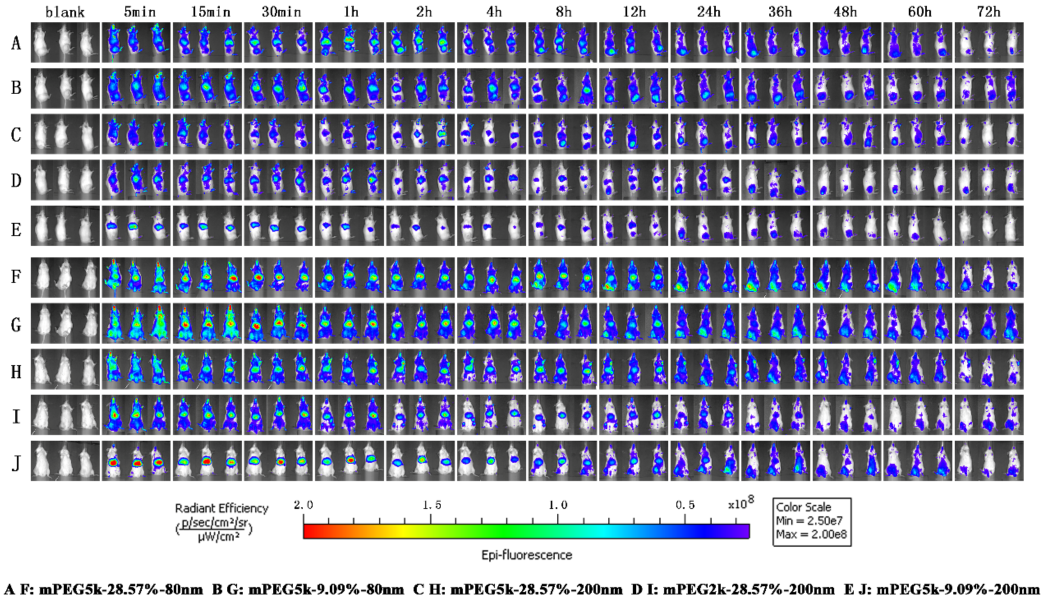

Figure S2. Whole animal imaging of S180 tumor bearing mice after intravenous administration of mPEG-PCL nanoparticles at the leteral position (A-E) and supine position (F-J).

Table S3. Physiological parameters used in the PBPK model for NPs in mice[1-3]

Parameter（unit）	Value

body weight (kg)	0.04[a]
cardiac output(L/h/kg$^{0.75}$)	16.5
**Blood flow to organ (fraction of cardiac output, unitless)**	
Heart	0.066
Liver	0.161
Spleen	0.01125
Lung	1
Kidney	0.091
Tumor	0.0125
**Organ volumes (fraction of body weight, unitless)**	
Heart	0.005
Liver	0.0549
Spleen	0.005
Lung	0.007
Kidney	0.0167
Tumor	0.055[a]
Blood	0.085
**Volume fraction of interstitial tissue in organs (unitless)**	
Heart	0.143
Liver	0.26
Spleen	0.2
Lung	0.3
Kidney	0.34
Tumor	0.38
Rest of body	0.21
**Volume fraction of blood in organs (unitless)**	
Heart	0.34[b]
Liver	0.31
Spleen	0.17
Lung	0.5
Kidney	0.24
Tumor	0.035[b]
Rest of body	0.04

a. Data are measured experimentally

b. Data are obtained by fitting in the software based on *in vivo* observations

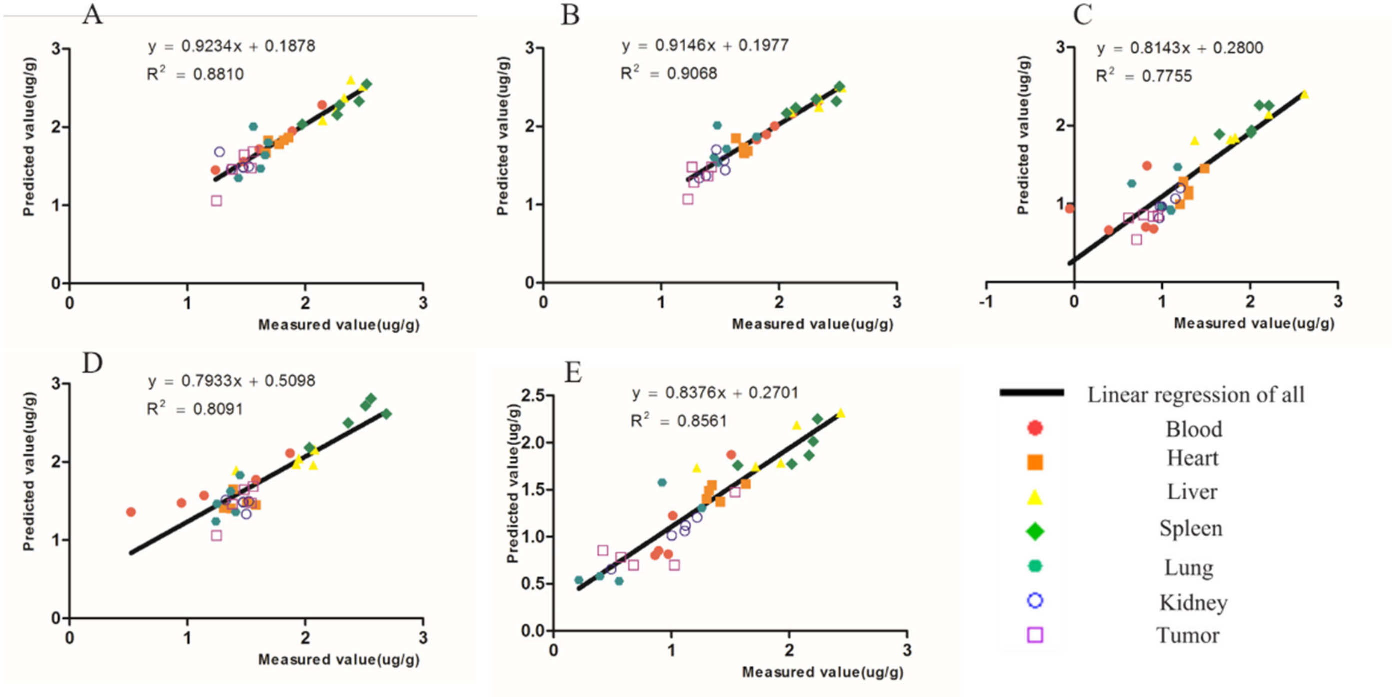

Figure S3. Goodness-of-fit plot of the linear regression analysis of model predictions and experimental data after intravenous injection for model calibration. Measured values are from our own experimental data. mPEG5k-9.09%-80nm NPs (A), mPEG5k-28.57%-80nm NPs (B), mPEG5k-9.09%-200nm NPs (C), mPEG5k-28.57%-200nm NPs (D) and mPEG2k-28.57%-200nm NPs (E).

Table S4. Relative sensitive analyses for the parameters of mPEG5k-9.09%-80nm NPs

Parameters	Blood	Heart	Liver	Spleen	Lung	Kidney	Tumor	Body
**BW**	**-0.98**	**-0.98**	**-1.00**	**-0.99**	**-0.99**	**-0.99**	**-0.99**	**-0.98**
**IV**	**1.00**	**0.98**	**1.00**	**1.02**	**1.00**	**0.99**	**0.99**	**1.01**
**VLC**	-0.17	-0.18	-0.19	-0.16	-0.17	-0.19	-0.18	-0.17
**VBloodC**	-0.01	0.00	-0.02	0.00	-0.01	-0.03	-0.02	0.00
**Pt**	0.00	0.00	0.00	0.00	0.00	0.00	0.00	0.00
**PACt**	0.00	0.00	0.00	0.00	0.00	0.00	0.00	0.00
**KupLumax**	0.00	0.00	0.00	0.00	0.36	0.00	0.00	0.00
**KupLu50**	0.00	0.00	0.00	0.00	0.00	0.00	0.00	0.00
**KupLun**	0.00	0.00	0.00	0.00	0.00	0.00	0.00	0.00
**KoutLu**	0.00	0.00	0.00	0.00	-0.34	0.00	0.00	0.01
**KupHmax**	0.00	**0.80**	-0.02	0.00	0.00	-0.01	-0.01	0.00
**KupH50**	0.00	0.00	0.00	0.00	0.00	0.00	0.00	0.00
**KupHn**	0.00	0.06	0.00	0.00	0.00	0.00	0.00	0.00
**KoutH**	0.01	-0.37	0.00	0.00	0.00	0.00	0.00	0.01
**KupTmax**	-0.04	-0.06	-0.05	-0.03	-0.04	-0.04	**0.90**	-0.04
**KupT50**	0.00	0.00	0.00	0.00	0.00	0.00	0.00	0.00
**KupTn**	0.00	0.00	0.00	0.00	0.00	0.00	0.01	0.00
**KoutT**	0.04	0.06	0.02	0.05	0.04	0.03	**-0.79**	0.05
**KupLmax**	-0.19	-0.18	**0.71**	-0.19	-0.20	-0.21	-0.21	-0.19
**KupL50**	0.00	0.00	0.00	0.00	0.00	0.00	0.00	0.00
**KupLn**	0.00	0.00	0.00	0.00	0.00	0.00	0.00	0.00
**KoutL**	0.19	0.24	**-0.74**	0.21	0.20	0.20	0.20	0.20
**KupSmax**	-0.01	0.00	-0.02	**0.94**	-0.01	-0.03	-0.02	-0.01
**KupS50**	0.00	0.00	0.00	0.00	0.00	0.00	0.00	0.00
**KupSn**	0.00	0.00	0.00	0.00	0.00	0.00	0.00	0.00
**KoutS**	0.02	0.00	0.00	**-0.91**	0.01	0.01	0.01	0.02
**KupKmax**	0.00	0.00	0.00	0.00	0.00	**0.67**	0.00	0.00
**KupK50**	0.00	0.00	0.00	0.00	0.00	-0.01	0.00	0.00
**KupKn**	0.00	0.00	0.00	0.00	0.00	0.05	0.00	0.00
**KoutK**	0.00	0.00	0.00	0.00	0.00	-0.36	0.00	0.01
**KupBomax**	**-0.56**	**-0.55**	**-0.57**	**-0.56**	**-0.57**	**-0.55**	**-0.55**	0.34
**KupBo50**	0.07	0.06	0.07	0.08	0.07	0.07	0.07	-0.04
**KupBon**	-0.12	-0.12	-0.14	-0.13	-0.13	-0.12	-0.12	0.08
**KoutBo**	0.36	0.31	0.36	0.35	0.36	0.30	0.32	-0.20

Table S5. Relative sensitive analyses for the parameters of mPEG5k-28.57%-80nm NPs

Parameters	Blood	Heart	Liver	Spleen	Lung	Kidney	Tumor	Body
**BW**	**-1.00**	**-1.02**	**-0.98**	**-0.98**	**-0.92**	**-0.99**	**-1.00**	**-1.00**
**IV**	**0.50**	**0.51**	**0.51**	**0.51**	**0.55**	0.49	**0.50**	0.49
**VLC**	-0.20	-0.17	-0.18	-0.19	-0.18	-0.21	-0.21	-0.19
**VBloodC**	-0.10	-0.09	-0.09	-0.09	-0.09	-0.12	-0.11	-0.10
**Pt**	0.00	0.00	0.00	0.00	0.00	0.00	0.00	0.00
**PACt**	0.00	0.00	0.00	0.00	0.00	0.00	0.00	0.00
**KupLumax**	0.00	0.00	0.00	0.00	0.09	0.00	0.00	0.00
**KupLu50**	0.00	0.00	0.00	0.00	0.00	0.00	0.00	0.00
**KupLun**	0.00	0.00	0.00	0.00	0.00	0.00	0.00	0.00
**KoutLu**	0.00	0.00	0.00	0.00	0.00	0.00	0.00	0.00
**KupHmax**	0.00	**0.60**	0.00	0.00	0.00	-0.01	0.00	-0.01
**KupH50**	0.00	0.00	0.00	0.00	0.00	0.00	0.00	0.00
**KupHn**	0.00	0.00	0.00	0.00	0.00	0.00	0.00	0.00
**KoutH**	0.00	-0.17	0.00	0.00	0.00	0.00	0.00	0.00
**KupTmax**	-0.05	0.00	-0.02	-0.02	0.00	-0.03	**0.73**	-0.03
**KupT50**	0.00	0.00	0.00	0.00	0.00	0.00	0.00	0.00
**KupTn**	0.00	0.00	0.00	0.00	0.00	0.00	0.00	0.00
**KoutT**	0.00	0.00	0.02	0.02	0.09	0.01	**-0.69**	0.01
**KupLmax**	-0.20	-0.17	**0.65**	-0.19	-0.18	-0.21	-0.21	-0.20
**KupL50**	0.00	0.00	0.00	0.00	0.00	0.00	0.00	0.00
**KupLn**	0.00	0.00	0.00	0.00	0.00	0.00	0.00	0.00
**KoutL**	0.20	0.17	**-0.65**	0.21	0.27	0.19	0.19	0.19
**KupSmax**	-0.05	0.00	0.00	**0.82**	0.00	-0.01	-0.02	-0.03
**KupS50**	0.00	0.00	0.00	0.00	0.00	0.00	0.00	0.00
**KupSn**	0.00	0.00	0.00	0.00	0.00	0.00	0.00	0.00
**KoutS**	0.00	0.00	0.02	**-0.86**	0.09	0.01	0.02	0.01
**KupKmax**	0.00	0.00	0.00	0.00	0.00	0.13	0.00	0.00
**KupK50**	0.00	0.00	0.00	0.00	0.00	-0.01	0.00	0.00
**KupKn**	0.00	0.00	0.00	0.00	0.00	0.00	0.00	0.00
**KoutK**	0.00	0.00	0.00	0.00	0.00	-0.07	0.00	0.00
**KupBomax**	-0.45	-0.43	-0.43	-0.42	-0.37	-0.43	-0.42	0.35
**KupBo50**	0.05	0.09	0.07	0.07	0.09	0.06	0.06	-0.05
**KupBon**	-0.10	-0.09	-0.07	-0.07	0.00	-0.07	-0.08	0.05
**KoutBo**	0.30	0.26	0.31	0.33	0.37	0.30	0.31	-0.27

Table S6. Relative sensitive analyses for the parameters of mPEG5k-9.09%-200nm NPs

Parameters	Blood	Heart	Liver	Spleen	Lung	Kidney	Tumor	Body
**BW**	**1.01**	**1.00**	**1.01**	**0.99**	**0.97**	**1.00**	**1.02**	**0.99**
**IV**	**1.01**	**1.00**	**1.01**	**0.99**	**0.97**	**1.00**	**1.02**	**0.99**
**VLC**	-0.16	-0.16	-0.17	-0.14	-0.13	-0.13	-0.12	-0.14
**VBloodC**	0.08	0.06	0.06	0.05	0.04	0.07	0.06	0.06
**Pt**	0.00	0.00	0.00	0.00	0.00	0.00	0.00	0.00
**PACt**	0.00	0.00	0.00	0.00	0.00	0.00	0.00	0.00
**KupLumax**	0.00	0.00	0.00	0.00	**0.71**	0.00	0.00	0.00
**KupLu50**	0.00	0.00	0.00	0.00	0.00	0.00	0.00	0.00
**KupLun**	0.00	0.00	0.00	0.00	0.00	0.00	0.00	0.00
**KoutLu**	0.00	0.00	0.00	0.00	**-0.71**	0.00	0.00	0.00
**KupHmax**	0.00	**0.90**	0.00	0.00	0.00	0.00	0.00	-0.02
**KupH50**	0.00	-0.04	0.00	0.00	0.00	0.00	0.00	0.00
**KupHn**	0.00	0.06	0.00	0.00	0.00	0.00	0.00	0.00
**KoutH**	0.00	-0.43	0.00	0.00	0.00	0.00	0.00	0.00
**KupTmax**	0.00	-0.02	0.00	-0.05	-0.04	0.00	**0.96**	-0.02
**KupT50**	0.00	0.00	0.00	0.00	0.00	0.00	-0.06	0.00
**KupTn**	0.00	0.00	0.00	0.00	0.00	0.00	0.18	0.00
**KoutT**	0.00	0.02	0.00	0.00	0.00	0.03	**-0.84**	0.00
**KupLmax**	-0.16	-0.20	**0.79**	-0.19	-0.18	-0.20	-0.18	-0.18
**KupL50**	0.00	0.00	0.00	0.00	0.00	0.00	0.00	0.00
**KupLn**	0.00	-0.02	0.06	-0.05	-0.04	0.00	0.00	-0.02
**KoutL**	0.16	0.18	**-0.79**	0.19	0.18	0.20	0.18	0.18
**KupSmax**	0.00	-0.02	0.00	**0.94**	-0.04	0.00	0.00	-0.02
**KupS50**	0.00	0.00	0.00	0.00	0.00	0.00	0.00	0.00
**KupSn**	0.00	0.00	0.00	0.00	0.00	0.00	0.00	0.00
**KoutS**	0.00	0.02	0.00	**-0.94**	0.00	0.03	0.00	0.02
**KupKmax**	0.00	0.00	0.00	0.00	0.00	**0.90**	0.00	0.00
**KupK50**	0.00	0.00	0.00	0.00	0.00	0.00	0.00	0.00
**KupKn**	0.00	0.00	0.00	0.00	0.00	0.07	0.00	0.00
**KoutK**	0.00	0.00	0.00	0.00	0.00	-0.47	0.00	0.00
**KupBomax**	**-0.70**	**-0.70**	**-0.73**	**-0.70**	**-0.71**	**-0.70**	**-0.72**	0.24
**KupBo50**	0.08	0.06	0.06	0.05	0.04	0.07	0.06	-0.02
**KupBon**	-0.16	-0.14	-0.11	-0.14	-0.13	-0.13	-0.12	0.04
**KoutBo**	**0.62**	**0.59**	**0.62**	**0.61**	**0.62**	**0.60**	**0.66**	-0.22

Table S7. Relative sensitive analyses for the parameters of mPEG5k-28.57%-200nm NPs

Parameters	Blood	Heart	Liver	Spleen	Lung	Kidney	Tumor	Body
**BW**	**-0.97**	**-0.99**	**-0.99**	**-0.99**	**-1.00**	**-0.98**	**-1.00**	**-1.00**
**IV**	**1.00**	**0.99**	**0.99**	**0.99**	**1.00**	**1.01**	**1.00**	**0.98**
**VLC**	-0.17	-0.19	-0.17	-0.20	-0.20	-0.21	-0.19	-0.12
**VBloodC**	-0.02	-0.01	-0.06	-0.03	-0.03	-0.03	-0.03	0.02
**Pt**	0.00	0.00	0.00	0.00	0.00	0.00	0.00	0.00
**PACt**	0.00	0.00	0.00	0.00	0.00	0.00	0.00	0.00
**KupLumax**	0.00	0.00	0.00	0.00	0.33	0.00	0.00	0.00
**KupLu50**	0.00	0.00	0.00	0.00	0.00	0.00	0.00	0.00
**KupLun**	0.00	0.00	0.00	0.00	0.00	0.00	0.00	0.00
**KoutLu**	0.00	0.00	0.00	0.00	-0.33	0.00	0.00	0.00
**KupHmax**	0.00	**0.79**	0.00	-0.01	0.00	0.00	0.00	-0.02
**KupH50**	0.00	-0.10	0.00	0.00	0.00	0.00	0.00	0.00
**KupHn**	0.00	0.10	0.00	0.00	0.00	0.00	0.00	0.00
**KoutH**	0.02	**-0.66**	0.00	0.00	0.00	0.00	0.00	0.00
**KupTmax**	-0.02	-0.04	-0.06	-0.04	-0.03	-0.03	**0.90**	-0.05
**KupT50**	0.02	0.00	0.00	0.00	0.00	0.00	-0.09	0.00
**KupTn**	0.00	-0.01	0.00	-0.01	-0.03	0.00	0.22	-0.02
**KoutT**	0.00	-0.01	0.00	-0.01	-0.03	0.00	0.22	-0.02
**KupLmax**	-0.20	-0.22	**0.72**	-0.21	-0.23	-0.23	-0.22	-0.16
**KupL50**	0.00	0.00	0.00	0.00	0.00	0.00	0.00	0.00
**KupLn**	0.00	0.00	0.00	-0.01	0.00	0.00	0.00	-0.02
**KoutL**	0.20	0.19	**-0.72**	0.18	0.17	0.21	0.19	0.14
**KupSmax**	0.20	0.19	**-0.72**	0.18	0.17	0.21	0.19	0.14
**KupS50**	0.02	0.00	0.00	-0.04	0.00	0.00	0.00	0.00
**KupSn**	0.00	-0.01	0.00	0.21	-0.03	-0.02	-0.03	-0.02
**KoutS**	0.07	0.07	0.06	**-0.88**	0.07	0.07	0.06	0.05
**KupKmax**	0.00	0.00	0.00	0.00	0.00	**0.84**	0.00	0.00
**KupK50**	0.00	0.00	0.00	0.00	0.00	-0.02	0.00	0.00
**KupKn**	0.00	0.00	0.00	0.00	0.00	0.09	0.00	0.00
**KoutK**	0.00	0.00	0.00	0.00	0.00	-0.47	0.00	0.00
**KupBomax**	-0.47	**-0.53**	**-0.50**	**-0.50**	**-0.50**	-0.44	**-0.53**	0.28
**KupBo50**	0.30	0.31	0.28	0.29	0.27	0.28	0.31	-0.19
**KupBon**	0.20	0.22	0.17	0.18	0.17	0.23	0.19	-0.12
**KoutBo**	0.30	0.29	0.28	0.28	0.27	0.23	0.31	-0.16

Table S8. Relative sensitive analyses for the parameters of mPEG5k-28.57%-200nm NPs

Parameters	Blood	Heart	Liver	Spleen	Lung	Kidney	Tumor	Body
**BW**	**-0.99**	**-0.99**	**-0.96**	**-1.01**	**-0.98**	**-0.99**	**-1.03**	**-0.98**
**IV**	**1.04**	**1.00**	**1.02**	**1.01**	**0.98**	**0.99**	**1.03**	**1.00**
**VLC**	-0.10	-0.13	-0.13	-0.18	-0.10	-0.15	-0.16	-0.10
**VBloodC**	0.05	0.03	0.06	0.00	0.00	0.03	0.00	0.06
**Pt**	0.00	0.00	0.00	0.00	0.00	0.00	0.00	0.00
**PACt**	0.00	0.00	0.00	0.00	0.00	0.00	0.00	0.00
**KupLumax**	0.00	0.00	0.00	0.00	0.00	0.00	0.00	0.00
**KupLu50**	0.00	0.00	0.00	0.00	0.00	0.00	0.00	0.00
**KupLun**	0.00	0.00	0.00	0.00	0.00	0.00	0.00	0.00
**KoutLu**	0.00	0.00	0.00	0.00	0.00	0.00	0.00	0.00
**KupHmax**	0.00	**0.89**	0.00	0.00	0.00	0.00	0.00	0.00
**KupH50**	0.00	-0.03	0.00	0.00	0.00	0.00	0.00	0.00
**KupHn**	0.00	0.06	0.00	0.00	0.00	0.00	0.00	0.00
**KoutH**	0.05	**-0.79**	0.00	0.00	0.00	0.00	0.00	0.02
**KupTmax**	0.00	-0.02	0.00	0.00	0.00	0.00	**0.95**	0.00
**KupT50**	0.00	0.00	0.00	0.00	0.00	0.00	-0.08	0.00
**KupTn**	0.00	0.00	0.00	0.00	0.00	0.00	0.16	0.00
**KoutT**	0.05	0.02	0.00	0.00	0.00	0.00	**-0.87**	0.02
**KupLmax**	-0.16	-0.17	**0.83**	-0.18	-0.20	-0.18	-0.16	-0.14
**KupL50**	0.05	0.02	0.00	0.00	0.00	0.00	0.00	0.00
**KupLn**	0.00	-0.02	0.06	0.00	0.00	-0.03	0.00	0.00
**KoutL**	0.21	0.16	**-0.76**	0.18	0.20	0.18	0.16	0.15
**KupSmax**	0.00	-0.02	0.00	**0.95**	0.00	-0.03	0.00	0.00
**KupS50**	0.00	0.00	0.00	0.00	0.00	0.00	0.00	0.00
**KupSn**	0.00	0.00	0.00	0.00	0.00	0.00	0.00	0.00
**KoutS**	0.05	0.02	0.00	**-0.95**	0.00	0.00	0.00	0.02
**KupKmax**	0.00	0.00	0.00	0.00	0.00	**0.87**	0.00	0.00
**KupK50**	0.00	0.00	0.00	0.00	0.00	-0.03	0.00	0.00
**KupKn**	0.00	0.00	0.00	0.00	0.00	0.09	0.00	0.00
**KoutK**	0.00	0.00	0.00	0.00	0.00	-0.45	0.00	0.00
**KupBomax**	**-0.68**	**-0.70**	**-0.70**	**-0.71**	**-0.69**	**-0.66**	**-0.71**	0.21
**KupBo50**	0.10	0.08	0.06	0.06	0.10	0.09	0.08	-0.02
**KupBon**	-0.05	-0.06	-0.06	-0.06	-0.10	-0.06	-0.08	0.02
**KoutBo**	**0.62**	**0.59**	**0.64**	**0.59**	**0.59**	**0.54**	**0.63**	-0.17

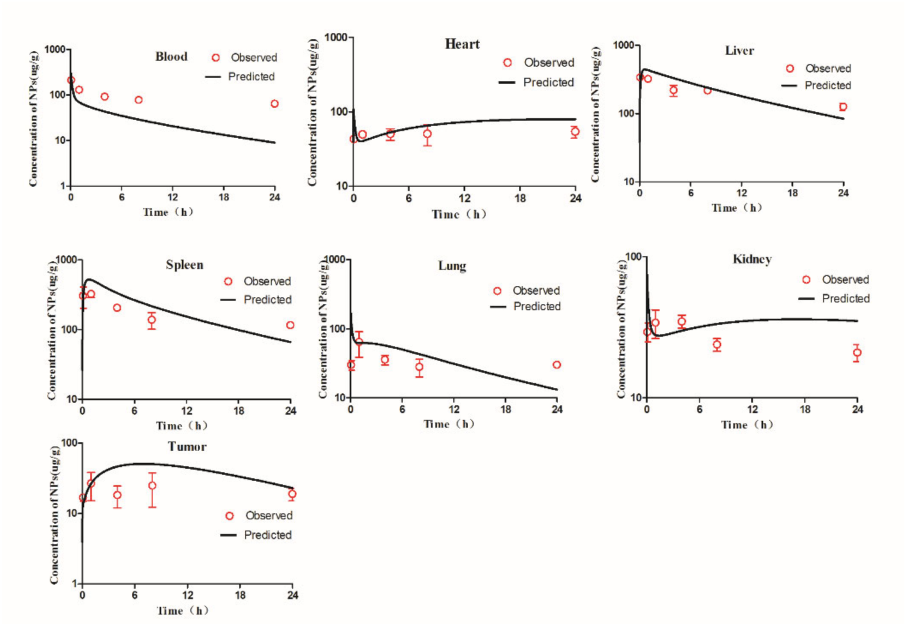

Figure S4. The simulated and experimentally measured concentration of mPEG5k-9.09%-80nm NPs in mice blood and tissues after intravenous injection. Simulation results are represented by solid lines in each panel, and the mean values of measured data are represented by red circles. Error bars represent standard deviation of experimentally measured data.

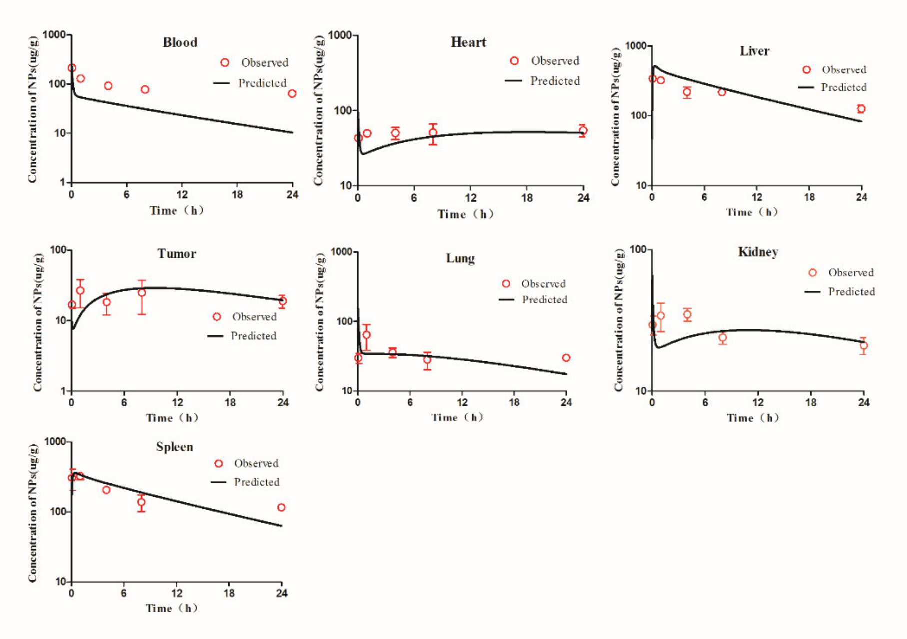

Figure S5. The simulated and experimentally measured concentration of mPEG5k-28.57%-80nm NPs in mice blood and tissues after intravenous injection.

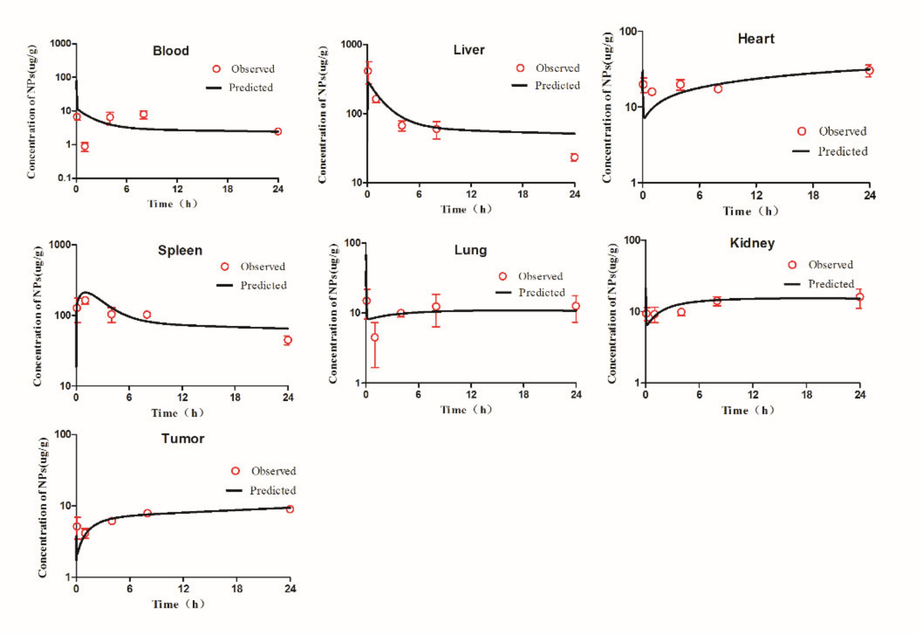

Figure S6. The simulated and experimentally measured concentration of mPEG5k-9.09%-200nm NPs in mice blood and tissues after intravenous injection.

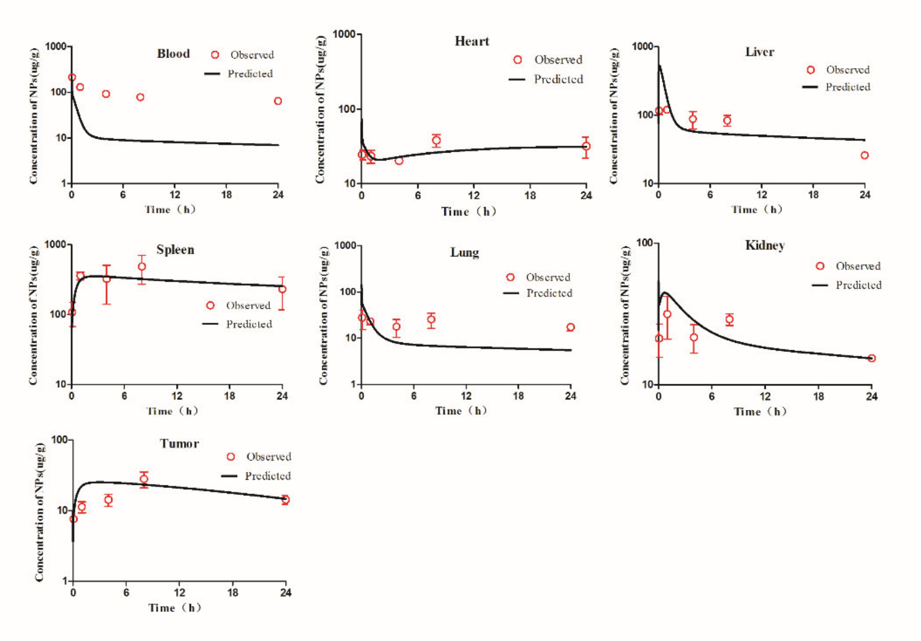

Figure S7. The simulated and experimentally measured concentration of mPEG5k-28.57%-200nm NPs in mice blood and tissues after intravenous injection.

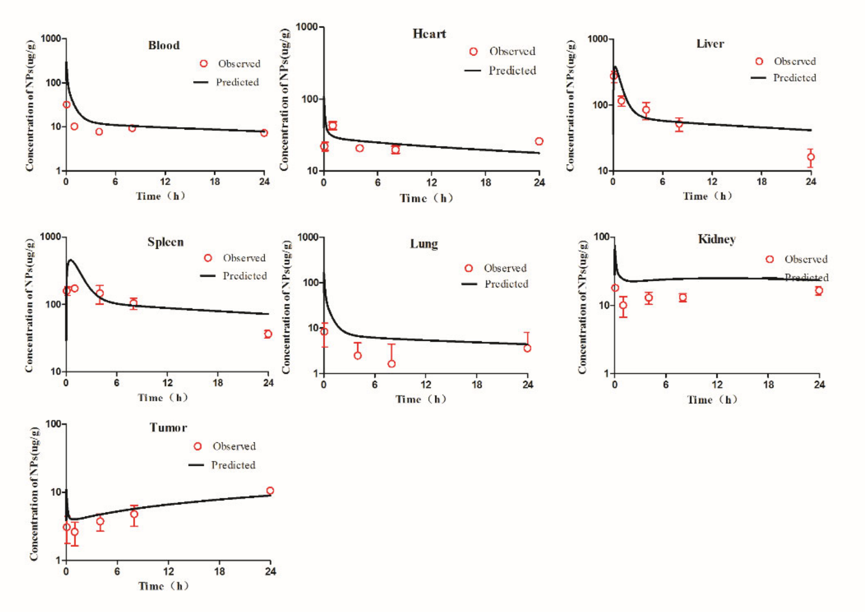

Figure S8. The simulated and experimentally measured concentration of mPEG2k -28.57%-200nm NPs in mice blood and tissues after intravenous injection.

Goodness of fit of the EPR-PBPK model for NPs

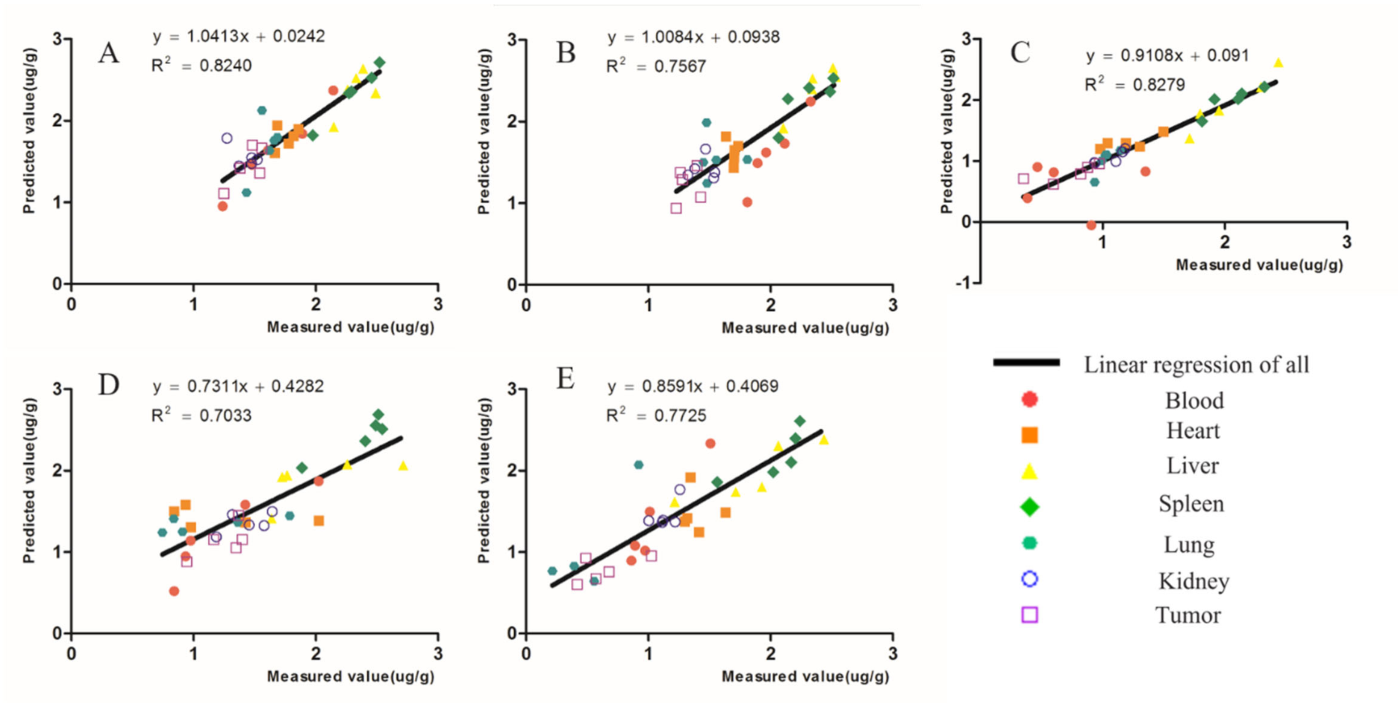

Figure S9. Goodness-of-fit plot of the linear regression analysis of model predictions and experimental data after intravenous injection for model calibration. Measured values are from our own experimental data. mPEG5k-9.09%-80nm NPs (A), mPEG5k-28.57%-80nm NPs (B), mPEG5k-9.09%-200nm NPs (C), mPEG5k-28.57%-200nm NPs (D) and mPEG2k-28.57%-200nm NPs (E)

Mass balance equations of the PBPK model

Mass balance equations

For venous blood

$$V_{V\_b}\frac{dCV_{Vb}}{dt} = Q_H CV_H + Q_L CV_L + Q_S CV_L + Q_K CV_K + Q_T CV_T + Q_{Bo} CV_{Bo} - Q_{Lu} CV_{Vb}$$

For arterial blood

$$V_A \frac{dC_A}{dt} = Q_{Lu}(CV_{Lu} - C_A)$$

For lung

For vascular space

$$V_{V\_Lu}\frac{dCV_{Lu}}{dt} = Q_{Lu}(C_{Vb} - CV_{Lu}) - PA_{Lu}CV_{Lu} + \frac{PA_{Lu}C_{T\_Lu}}{P_{Lu}} - k_{up\_Lu}CV_{Lu}V_{V\_Lu} + k_{release\_Lu}A_{PC\_Lu}$$

For tissue (interstitial space)

$$V_{T\_Lu}\frac{dC_{T\_Lu}}{dt} = PA_{Lu}CV_{Lu} - \frac{PA_{Lu}C_{T\_Lu}}{P_{Lu}}$$

For phagocytic cell in lung

$$\frac{dA_{PC\_Lu}}{dt} = k_{up\_Lu}CV_{Lu}V_{V\_Lu} - k_{release\_Lu}A_{PC\_Lu}$$

PCL-P2 NPs concentration in the lung is shown by:

$$dC_{Total\_Lu} = \frac{CV_{Lu}V_{V\_Lu} + C_{T\_Lu}V_{T\_Lu} + A_{PC\_Lu}}{V_{Lu}}$$

For liver

For vascular space

$$V_{V\_L}\frac{dCV_L}{dt} = Q_L\mathrm{CA} + Q_S CV_S - (Q_L + Q_S)CV_L - PA_L CV_L + \frac{PA_L C_{T\_L}}{P_L} - k_{up\_L}CV_L V_{V\_L} + k_{release\_L}A_{PC\_L}$$

For tissue (interstitial space)

$$V_{T\_L}\frac{dC_{T\_L}}{dt} = PA_L CV_L - \frac{PA_L C_{T\_L}}{P_L}$$

For phagocytic cell in liver

$$\frac{dA_{PC\_L}}{dt} = k_{up\_L}CV_L V_{V\_L} - k_{release\_L}A_{PC\_L}$$

PCL-P2 NPs concentration in the liver is shown by:

$$dC_{Total\_L} = \frac{CV_L V_{V\_L} + C_{T\_L}V_{T\_L} + A_{PC\_L}}{V_L}$$

For spleen

For vascular space

$$V_{V\_S}\frac{dCV_S}{dt} = Q_S(\mathrm{CA} - CV_S) - PA_S CV_S + \frac{PA_S C_{T\_S}}{P_S} - k_{up\_S}CV_S V_{V\_S} + k_{release\_S}A_{PC\_S}$$

For tissue (interstitial space)

$$V_{T\_S}\frac{dC_{T\_S}}{dt} = PA_S CV_S - \frac{PA_S C_{T\_S}}{P_S}$$

For phagocytic cell in spleen

$$\frac{dA_{PC\_S}}{dt} = k_{up\_S} CV_S V_{V\_S} - k_{release\_S} A_{PC\_S}$$

PCL-P2 NPs concentration in the spleen is shown by:

$$dC_{Total\_S} = \frac{CV_S V_{V\_S} + C_{T\_S} V_{T\_S} + A_{PC\_S}}{V_S}$$

For kidney

$$V_{V\_K}\frac{dCV_K}{dt} = Q_K(\mathrm{CA} - CV_K) - PA_K CV_K + \frac{PA_K C_{T\_K}}{P_K} - k_{up\_K} CV_K V_{V\_K} + k_{release\_K} A_{PC\_K}$$

For tissue (interstitial space)

$$V_{T\_K}\frac{dC_{T\_K}}{dt} = PA_K CV_K - \frac{PA_K C_{T\_K}}{P_K}$$

For phagocytic cell in kidney

$$\frac{dA_{PC\_K}}{dt} = k_{up\_K} CV_K V_{V\_K} - k_{release\_K} A_{PC\_K}$$

PCL-P2 NPs concentration in the kidney is shown by:

$$dC_{Total\_K} = \frac{CV_K V_{V\_K} + C_{T\_K} V_{T\_K} + A_{PC\_K}}{V_K}$$

For heart

For vascular space

$$V_{V\_H}\frac{dCV_H}{dt} = Q_H(\mathrm{CA} - CV_H) - PA_H CV_H + \frac{PA_H C_{T\_H}}{P_H} - k_{up\_H} CV_H V_{V\_H} + k_{release\_H} A_{PC\_H}$$

For tissue (interstitial space)

$$V_{T\_H}\frac{dC_{T\_H}}{dt} = PA_H CV_H - \frac{PA_H C_{T\_H}}{P_H}$$

For phagocytic cell in heart

$$\frac{dA_{PC\_H}}{dt} = k_{up\_H} CV_H V_{V\_H} - k_{release\_H} A_{PC\_H}$$

PCL-P2 NPs concentration in the heart is shown by:

$$dC_{Total\_H} = \frac{CV_H V_{V\_H} + C_{T\_H} V_{T\_H} + A_{PC\_H}}{V_H}$$

For tumor

For vascular space

$$V_{V\_T}\frac{dCV_T}{dt} = Q_T(\mathrm{CA} - CV_T) - PA_T CV_T + \frac{PA_T C_{T\_T}}{P_T} - k_{up\_T} CV_T V_{V\_T} + k_{release\_T} A_{PC\_T}$$

For tissue (interstitial space)

$$V_{T\_T}\frac{dC_{T\_T}}{dt} = PA_T CV_T - \frac{PA_T C_{T\_T}}{P_T}$$

For phagocytic cell in tumor

$$\frac{dA_{PC\_T}}{dt} = k_{up\_T} CV_T V_{V\_T} - k_{release\_T} A_{PC\_T}$$

PCL-P2 NPs concentration in the tumor is shown by:

$$\boldsymbol{dC_{Total\_T} = \frac{CV_T V_{V\_T} + C_{T\_T} V_{T\_T} + A_{PC\_T}}{V_T}}$$

For rest of body

$$\boldsymbol{V_{V\_Bo} \frac{dCV_{Bo}}{dt} = Q_{Bo}(\mathrm{CA} - CV_{Bo}) - PA_{Bo}CV_{Bo} + \frac{PA_{Bo}C_{T\_Bo}}{P_{Bo}} - k_{up\_Bo}CV_{Bo}V_{V\_Bo} + k_{release\_Bo}A_{PC\_Bo}}$$

For tissue (interstitial space)

$$V_{T\_Bo} \frac{dC_{T\_Bo}}{dt} = PA_{Bo}CV_{Bo} - \frac{PA_{Bo}C_{T\_Bo}}{P_{Bo}}$$

For phagocytic cell in rest of body

$$\frac{dA_{PC\_Bo}}{dt} = k_{up\_Bo}CV_{Bo}V_{V\_Bo} - k_{release\_Bo}A_{PC\_Bo}$$

PCL-P2 NPs concentration in the rest of body is shown by:

$$dC_{Total\_Bo} = \frac{CV_{Bo}V_{V\_Bo} + C_{T\_Bo}V_{T\_Bo} + A_{PC\_Bo}}{V_{Bo}}$$

The code in the PBPK model for IV injection of NPs

The model for IV injection of PCL-P2 NPs

```
METHOD RK4

STARTTIME= 0

STOPTIME=24

DTMAX = 0.01

DTOUT =0.1

; Physiological constants (kg)

BW = 0.04 ; body weight

; Dose (ul)

IV = 200

; Organ volumes (fraction of body weight)

VLuC=0.007 ; Lung; Brown et al, 1997

VLC=0.0549 ; Liver; Brown et al, 1997

VSC=0.005 ; Spleen; Davies and Morris 1993

VKC=0.0167 ; Kidneys; Brown et al. 1997

VHC=0.005 ;    Lung; Brown et al, 1997

VTC=0.055 ;    Calculated from the data from literature

VBloodC=0.085 ; Blood; Davies and Morris 1993

VBoC=1-(VLC+VKC+VSC+VHC+VTC+VBloodC) ; Rest of body

; Organ volumes (L)

VBlood=VBloodC*BW

VLu=VLuC*BW ; Lung

VL=VLC*BW ; Liver

VS=VSC*BW ; Spleen

VK=VKC*BW ; Kidneys

VH=VHC*BW ; Heart

VT=VTC*BW ; Tumor

VA = 0.2*VBloodC*BW ; Arterial blood

VV = 0.8*VBloodC*BW ; Venous blood

VBo=VBoC*BW ; Rest of body
```

; Organ blood volumes (L)

VLuVES = 0.5*Vlu ; Lung; Brown et al, 1997

VLVES = 0.31*VL ; Liver; Brown et al, 1997

VSVES = 0.17*VS ; Spleen; Brown et al, 1997

VKVES = 0.24*VK ; Kidneys; Brown et al, 1997

VHVES =0.34*VH ; Heart fitted from the software

VTVES = 0.035*VT ; Tumor fitted from the software

VBoVES = 0.04*VBo ; Rest of body; Lin et al, 2015; Brown et al, 1997

; Organ tissue (interstitial) volumes (L)

VLuT = 0.3*Vlu ; Lung; Calculated from Davda et al, 2008

VLT = 0.26*VL ; Liver; Calculated from Davda et al, 2008

VST = 0.2*VS ; Spleen; Calculated from Davda et al, 2008

VKT = 0.34*VK ; Kidneys; Calculated from Davda et al, 2008

VHT = 0.143*VH ; Heart;Calculated from Davda et al, 2008

VTT = 0.38*VT ;Tumor;Calculated from Davda et al, 2008

VBoT = 0.21*VBo ; Rest of body; Calculated from Davda et al, 2008

; Blood flow rate (fraction of cardiac output)

QCC=16.5 ; Cardiac output constant; Brown et al, 1997

QLC=0.161 ; Liver; Brown et al, 1997.

QKC=0.091 ; Kidney; Brown et al, 1997

QSC=0.01125 ; Spleen; Lin et al, 2015; Davies and Morris, 1993

QHC=0.066 ; Heart; Brown et al, 1997

QTC=0.0125 ; Tumor;Calculated from Davda et al, 2008

QBoC=1-(QLC+QSC+QKC+QHC+QTC) ; Rest of body

; Cardiac output and regional blood flow (L/h)

QC=QCC*BW^0.75

QLu=QC ; Lung

QL=QLC*QC ; Liver

QS=QSC*QC ; Spleen

QK=QKC*QC ; Kidneys

QT=QTC*QC ;Tumor

QH=QHC*QC ; Heart

QBo=QBoC*QC ; Rest of body

; Distribution coefficients (PC), unitless

PLu =0.15 ; Lung

PL = 0.08 ; Liver

PS = 0.15 ; Spleen

PK = 0.15 ; Kidneys

PT = 0.15 ; Tumor

PH = 0.15 ; Heart

PBo = 0.15 ; Rest of body

; Diffusion limitation coefficient constants, unitless

PALuC = 0.001 ; Lung

PALC = 0.001 ; Liver

PASC = 0.03 ; Spleen

PAKC = 0.001 ; Kidneys

PATC= 0.001 ; Tumor

PAHC= 0.001 ; Heart

PABoC = 0.001 ; Rest of body

; Permeability coefficient-surface area cross-product

PALu = PALuC*QLu ; Lung

PAL = PALC*QL ; Liver

PAS = PASC*QS ; Spleen

PAH = PAHC*QH ; Heart

PAT = PATC*QT ; Tumor

PAK = PAKC*QK ; Kidneys

PABo = PABoC*QBo ; Rest of body

; Phagocytosis-related parameters; Lu, L, K, S, and Bo represent the lung, liver, kidneys, and rest of body, respectively

KupLumax=2.95062

KupLu50=0.0501613

KupLun=0.569474

KoutLu=1.99111e-8

KupLu=((KupLumax*time^KupLun)/(KupLu50^KupLun+time^KupLun))

KupHmax=6.2

KupH50 =150

KupHn=2.3e-4

KoutH=1.3e-9

KupH=((KupHmax*time^KupHn)/(KupH50^KupHn+time^KupHn))KupHmax=0.0026

KupTmax=6.3

KupT50=0.68

KupTn=0.0131083

KoutT=0.0034

KupT=((KupTmax*time^KupTn)/(KupT50^KupTn+time^KupTn))

KupLmax=14

KupL50=0.00956512

KupLn=160

KoutL=0.0257

KupL=((KupLmax*time^KupLn)/(KupL50^KupLn+time^KupLn))

KupSmax=170

KupS50=60

KupSn=0.14

KoutS=0.111701

KupS=((KupSmax*time^KupSn)/(KupS50^KupSn+time^KupSn))

KupKmax=4.5

KupK50 =52.9899

KupKn=0.0056163

KoutK=1.60941e-8

KupK=((KupKmax*time^KupKn)/(KupK50^KupKn+time^KupKn))

KupBomax=20.1296

```
KupBo50=0.034

KupBon=8.48464

KoutBo   =3.37e-6

KupBo=((KupBomax*time^KupBon)/(KupBo50^KupBon+time^KupBon))

; Urine and biliary excretion

Kurine=0.0012

Kbile=0.0012

; Blood compartment

; Venous blood concentration

d/dt (AV) = (QL*CVL+ QS*CVL+ QK*CVK+QT*CVT+QH*CVH + QBo*CVBo)-(QC*CV)

init AV = IV

CV = AV/VV

; Arterial blood concentration

d/dt (AA) = QC*(CVLu-CA)

init AA = 0

CA = AA/VA

CBlood=CA+CV

; Lung compartment; VES, T and RES represent blood vessels, tissue, and phagocytic cells

d/dt (ALuVES) = QLu*(CV-CVLu) - PALu*CVLu + (PALu*CLuT)/PLu+ KoutLu *ALuRES -
KupLu *ALuVES

init ALuVES = 0

CVLu = ALuVES/VLuVES

d/dt (ALuT) = PALu*CVLu - (PALu*CLuT)/PLu

init ALuT = 0

CLuT = ALuT/VLuT

d/dt (ALuRES) = KupLu *ALuVES - KoutLu *ALuRES

init ALuRES = 0

CLung = (ALuVES+ALuT+ALuRES)/VLu

; Liver compartment

d/dt (ALVES) = QL*CA+QS*CVS-(QL+QS)*CVL - PAL*CVL + (PAL*CLT)/PL + KoutL *ALRES
```

```
- KupL*ALVES - Kbile*CVL

init ALVES = 0

CVL = ALVES/VLVES

d/dt (Abile) = Kbile*CVL

init Abile = 0

d/dt (ALT) = PAL*CVL - (PAL*CLT)/PL

init ALT = 0

CLT = ALT/VLT

d/dt (ALRES) = KupL *ALVES - KoutL *ALRES

init ALRES = 0

CLiver = (ALVES+ALT+ALRES)/VL

; Spleen compartment

d/dt (ASVES) = QS*(CA-CVS) - PAS*CVS + (PAS*CST)/PS+ KoutS *ASRES - KupS *ASVES

init ASVES = 0

CVS = ASVES/VSVES

d/dt (AST) = PAS*CVS - (PAS*CST)/PS

init AST = 0

CST = AST/VST

d/dt (ASRES) = KupS *ASVES - KoutS *ASRES

init ASRES = 0

CSpleen = (ASVES+AST+ASRES)/VS

; Kidney compartment

d/dt (AKVES) = QK*(CA-CVK) - PAK*CVK + (PAK*CKT)/PK - Kurine*CVK+ KoutK *AKRES

- KupK *AKVES

init AKVES = 0

CVK = AKVES/VKVES

d/dt (Aurine) = Kurine*CVK

init Aurine = 0

d/dt (AKT) = PAK*CVK - (PAK*CKT)/PK

init AKT = 0
```

CKT = AKT/VKT

d/dt (AKRES) = KupK *AKVES - KoutK *AKRES

init AKRES = 0

CKidney = (AKVES+AKT+AKRES)/VK

; Heart compartment

d/dt (AHVES) = QH*(CA-CVH) - PAH*CVH + (PAH*CHT)/PH + KoutH *AHRES - KupH *AHVES

init AHVES = 0

CVH = AHVES/VHVES

d/dt (AHT) = PAH*CVH - (PAH*CHT)/PH

init AHT = 0

CHT = AHT/VHT

d/dt (AHRES) = KupH *AHVES - KoutH *AHRES

init AHRES = 0

CHeart = (AHVES+AHT+AHRES)/VH

;Tumor compartment

d/dt (ATVES) = QT*(CA-CVT) - PAK*CVT + (PAK*CTT)/PT + KoutT *ATRES - KupT *ATVES

init ATVES = 0

CVT = ATVES/VTVES

d/dt (ATT) = PAT*CVT - (PAT*CTT)/PT

init ATT = 0

CTT = ATT/VTT

d/dt (ATRES) = KupT *ATVES - KoutT *ATRES

init ATRES = 0

CTumor= (ATVES+ATT+ATRES)/VT

; Rest of body compartment

d/dt (ABoVES) = QBo*(CA-CVBo) - PABo*CVBo + (PABo*CBoT)/PBo + KoutBo *ABoRES - KupBo *ABoVES

init ABoVES = 0

CVBo = ABoVES/VBoVES

d/dt (ABoT) = PABo*CVBo - (PABo*CBoT)/PBo

init ABoT = 0

CBoT = ABoT/VBoT

d/dt (ABoRES) = KupBo *ABoVES - KoutBo *ABoRES

init ABoRES = 0

CBody = (ABoVES+ABoT+ABoRES)/VBo

References:

(1) Brown, R. P.; Delp, M. D.; Lindstedt, S. L.; Rhomberg, L. R.; Beliles, R. P.Physiological Parameter Values for Physiologically Based Pharmacokinetic Models. *Toxicol Ind Health* **1997**, *13*, 407-84.

(2) Davies, B.; Morris, T. Physiological Parameters in Laboratory Animals and Humans.*Pharm Res* **1993**, *10*, 1093-5.

(3) Davda, J. P.; Jain, M.; Batra, S. K.; Gwilt, P. R.; Robinson, D. H. A physiologically based pharmacokinetic (PBPK) model to characterize and predict the disposition of monoclonal antibody CC49 and its single chain Fv constructs.*International Immunopharmacology* **2008**, *8*, 401-413.
